# The mobile SAR signal N-hydroxypipecolic acid induces NPR1-dependent transcriptional reprogramming and immune priming

**DOI:** 10.1093/plphys/kiab166

**Published:** 2021-04-19

**Authors:** Ipek Yildiz, Melissa Mantz, Michael Hartmann, Tatyana Zeier, Jana Kessel, Corinna Thurow, Christiane Gatz, Patrick Petzsch, Karl Köhrer, Jürgen Zeier

**Affiliations:** 1 Department of Biology, Institute for Molecular Ecophysiology of Plants, Heinrich Heine University, Düsseldorf D-40225, Germany; 2 Department of Plant Molecular Biology and Physiology, Albrecht-von-Haller Institute for Plant Sciences, University of Göttingen, Göttingen D-37077, Germany; 3 Medical Faculty, Biological and Medical Research Center (BMFZ), Heinrich Heine University, Düsseldorf D-40225, Germany; 4 Cluster of Excellence on Plant Sciences (CEPLAS), Düsseldorf D-40225, Germany

## Abstract

N-hydroxypipecolic acid (NHP) accumulates in the plant foliage in response to a localized microbial attack and induces systemic acquired resistance (SAR) in distant leaf tissue. Previous studies indicated that pathogen inoculation of Arabidopsis (*Arabidopsis thaliana*) systemically activates SAR-related transcriptional reprogramming and a primed immune status in strict dependence of FLAVIN-DEPENDENT MONOOXYGENASE 1 (FMO1), which mediates the endogenous biosynthesis of NHP. Here, we show that elevations of NHP by exogenous treatment are sufficient to induce a SAR-reminiscent transcriptional response that mobilizes key components of immune surveillance and signal transduction. Exogenous NHP primes Arabidopsis wild-type and NHP-deficient *fmo1* plants for a boosted induction of pathogen-triggered defenses, such as the biosynthesis of the stress hormone salicylic acid (SA), accumulation of the phytoalexin camalexin and branched-chain amino acids, as well as expression of defense-related genes. NHP also sensitizes the foliage systemically for enhanced SA-inducible gene expression. NHP-triggered SAR, transcriptional reprogramming, and defense priming are fortified by SA accumulation, and require the function of the transcriptional coregulator NON-EXPRESSOR OF PR GENES1 (NPR1). Our results suggest that NPR1 transduces NHP-activated immune signaling modes with predominantly SA-dependent and minor SA-independent features. They further support the notion that NHP functions as a mobile immune regulator capable of moving independently of active SA signaling between leaves to systemically activate immune responses.

## Teaser

N-hydroxypipecolic acid functions as a mobile activator of systemic acquired resistance, induces NPR1-dependent transcriptional reprogramming, and primes plants for enhanced immune responses.

## Introduction

Phytopathogens must overcome several preformed and inducible defenses to cause disease in plants (Thordal-Christensen, 2003). To mount inducible defense responses, plants recognize extrinsic molecular patterns or pathogen effectors by immune receptor proteins (Zipfel, 2014). These include receptor-like protein (RLP) kinases (RLKs), RLPs, and nucleotide-binding site-leucine-rich repeat (NBS-LRR) type of resistance proteins (NLRs; Gust and Felix, 2014; Adachi et al., 2019). Pathogen recognition triggers plant immune signaling cascades that commonly result in increased expression of a battery of defense-related genes, biosynthesis of signal-active metabolites, accumulation of antimicrobial phytoalexins, cell wall fortifications, and the hypersensitive cell death response (Coll et al., 2011; Ahuja et al., 2012; Chezem et al., 2017; Liang and Zhou, 2018; Nobori et al., 2018).

Basal immune responses of unprepared plants are generally not effective enough to entirely prevent infection by well-adapted pathogens. However, a localized leaf inoculation can induce systemic acquired resistance (SAR) in the whole plant foliage (Shah and Zeier, 2013; Vlot et al., 2020). Plants with activated SAR show broad-spectrum immunity against further microbial infestation and are primed for a timely and boosted induction of defenses in response to pathogens (Jung et al., 2009; Návarová et al., 2012). The establishment of SAR requires an interplay of the two immune-regulatory metabolites salicylic acid (SA) and N-hydroxypipecolic acid (NHP), which both accumulate to substantial levels in inoculated and in distant, noninoculated leaves of pathogen-attacked plants (Hartmann et al., 2018; Hartmann and Zeier, 2019).

In the model plant Arabidopsis (*Arabidopsis thaliana*), stress-inducible SA is predominantly synthesized via the isochorismate pathway that includes plastidial conversion of chorismate to isochorismate by ISOCHORISMATE SYNTHASE1 (ICS1), transport of isochorismate to the cytosol via the MATE transporter ENHANCED DISEASE SUSCEPTIBILITY5 (EDS5), conjugation of isochorismate with glutamate by the GH3 acyl adenylase-family enzyme avrPphB Susceptible 3 (PBS3) in the cytosol, and breakdown of the resulting isochorismoyl-glutamate into SA (Nawrath and Métraux, 1999; Wildermuth et al., 2001; Rekhter et al., 2019; Torrens-Spence et al., 2019). SA relocates the transcriptional coactivator NON-EXPRESSOR OF PR GENES1 (NPR1) from the cytosol to the nucleus and binds to NPR1 to prompt increased expression of pathogenesis-related genes and immune activation (Mou et al., 2003; Wu et al., 2012; Ding et al., 2018; Ding and Ding, 2020).

The N-hydroxylated amino acid NHP has been identified as a plant natural product and immune signal much more recently (Hartmann et al., 2018). Arabidopsis as well as several other mono- and dicotyledonous plant species biosynthesize NHP in response to bacterial, fungal, and oomycete infection (Hartmann et al., 2018; Holmes et al., 2019; Schnake et al., 2020). NHP is produced from its direct metabolic precursor pipecolic acid (Pip) by an N-hydroxylation reaction catalyzed by FLAVIN-DEPENDENT MONOOXYGENASE 1 (FMO1; Chen et al., 2018; Hartmann et al., 2018), which itself is generated from L-Lys by consecutive transamination and reduction steps that are mediated by AGD2-LIKE DEFENSE RESPONSE PROTEIN1 (ALD1) and SAR-DEFICIENT4 (SARD4), respectively (Návarová et al., 2012; Ding et al., 2016; Hartmann et al. 2017; Xu et al., 2018). Arabidopsis *ald1* and *fmo1* mutant plants unable to accumulate NHP fail to establish pathogen-triggered SAR in Arabidopsis (Song et al., 2004; Mishina and Zeier, 2006). Whereas exogenous treatment with the NHP precursor Pip restored SAR in Pip-deficient *ald1*, it failed to do so in *fmo1* (Návarová et al., 2012). In contrast, application of NHP conferred SAR competency to both *ald1* and *fmo1* (Chen et al., 2018; Hartmann et al., 2018). Together, these studies indicate that NHP functions as a critical endogenous regulator of biologically induced SAR in Arabidopsis. SAR was also triggered in genetically engineered tomato that transiently expressed the Arabidopsis *ALD1* and *FMO1* genes (Holmes et al., 2019), and in several monocot and dicot species exogenously supplied with NHP (Schnake et al., 2020). This indicates a conserved function of NHP as a SAR-activating plant immune signal. Accumulating NHP is converted in planta to two distinct glucose conjugates, NHP-β-glucoside (NHPG) and NHP glucose ester (NHPGE; Chen et al., 2018; Hartmann and Zeier, 2018). Interestingly, SA and NHP share a common glycosyltransferase, UGT76B1, which converts both immune signals to their respective inactive β-glucosides (Bauer et al., 2021; Cai et al., 2021; Holmes et al., 2021; Mohnike et al., 2021).

The NHP biosynthetic pathway and the critical role of NHP as an endogenous activator of SAR have been profoundly elaborated in the past years. However, the mode of action of how NHP elevates plant immunity is not yet sufficiently understood. Different observations suggest that NHP functions as the long-sought-after mobile signal that travels from pathogen-inoculated to distant leaves in the course of SAR establishment. For example, NHP accumulated systemically in the leaf phloem sap of cucumber locally leaf-inoculated by bacterial pathogens (Schnake et al., 2020). Moreover, NHP exogenously applied to single Arabidopsis leaves was able to induce SAR in distant leaves (Chen et al., 2018; Schnake et al., 2020). Strictly dependent on the NHP synthase FMO1, exogenous treatment of Arabidopsis with the NHP precursor Pip induced a SAR-like transcriptional response and primed plants for enhanced pathogen-triggered immune responses (Bernsdorff et al., 2016; Hartmann et al., 2018). This indirectly suggested the possibility that NHP functions as a mediator of SAR-associated transcriptional reprogramming and defense priming. NHP induced a strong SAR only in plants capable of inducible SA biosynthesis, which indicates a tight interplay between NHP and SA in the activation of systemic immunity (Hartmann et al., 2018; Hartmann and Zeier, 2019).

In this study, we show that exogenously applied NHP is sufficient to induce upregulation of more than 1,500 SAR-related genes in Arabidopsis and primes plants for an enhanced pathogen-triggered activation of defense metabolism. Primed metabolic responses included the biosynthesis of SA, Pip, and branched-chain amino acids (BCAAs), as well as accumulation of the phytoalexin camalexin. NHP also conditioned Arabidopsis for effective SA- and pathogen-induced expression of defense-related genes. Notably, NHP-inducible SAR, transcriptional reprogramming, and immune priming strongly depended on the transcriptional coactivator NPR1. Our data further emphasize the function of NHP as a mobile SAR regulator, highlight positive interplay between NHP and SA in immune activation, and directly show that NHP mediates transcriptional reprogramming and defense priming during SAR.

## Results

Previous studies indicated that treatment of Arabidopsis Col-0 plants with a 1 mM NHP solution, either applied via the soil or sprayed on the leaf rosette, triggered a strong SAR response in the leaves (Chen et al., 2018; Hartmann et al., 2018; Schnake et al., 2020). Moreover, when individual leaves of Col-0 plants were treated with NHP, acquired resistance developed not only in the treated leaves but also in distant, systemic leaves (Chen et al., 2018; Schnake et al., 2020). SAR induction by soil application of NHP was greatly diminished in the *sid2/ics1* mutant that is unable to accumulate SA upon stress exposure, indicating that the NHP-triggered induction of a strong SAR response requires an intact SA biosynthetic pathway (Hartmann et al., 2018).

### Induction of SAR by NHP requires the transcriptional coregulator NPR1

**Figure 1:**
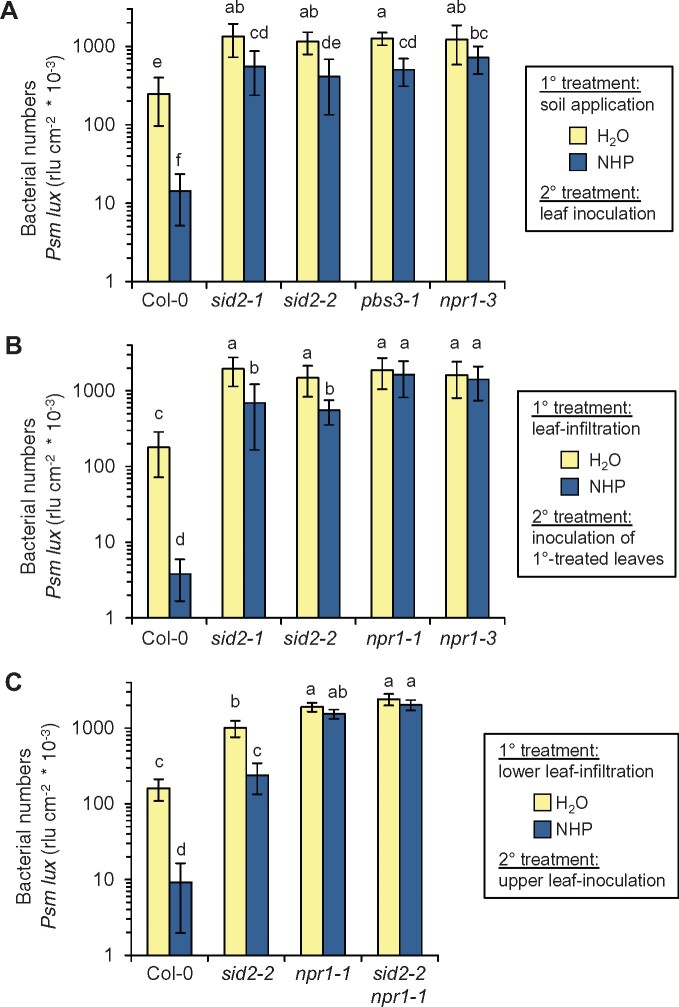
Arabidopsis SAR against *P. syringae* triggered by exogenous NHP is modest in the absence of inducible SA biosynthesis and requires NPR1. A, Individual Arabidopsis plants were supplied with 10 mL of an aqueous 1 mM NHP solution or with 10 mL of H_2_O via the cultivation soil (1° treatment). Three leaves of a plant were inoculated 1 d later with the bioluminescent *Psm lux* strain (OD_600_ = 0.001; 2° treatment). Bacterial numbers were determined at 2.5 dpi and expressed as rlus/cm^2^ leaf area (Hartmann et al., 2018). Bars indicate the mean ± sd of at least 12 biological replicates (*n* ≥ 12). B, Locally induced acquired resistance by foliar treatment with exogenous NHP. Three leaves of a plant were syringe-infiltrated with NHP solution (1 mM) or H_2_O (1° treatment). One day later, the same leaves were inoculated with *Psm lux* and bacterial numbers quantified at 2.5 dpi. Bars indicate the mean ± sd of at least 16 biological replicates (*n* ≥ 16). C, SAR by foliar treatment with exogenous NHP. Three lower leaves of a plant were syringe-infiltrated with 1 mM NHP or H_2_O (1° treatment). One day later, three upper, distant leaves were inoculated with *Psm lux* and bacterial numbers quantified at 2.5 dpi. Bars indicate the mean ± sd of at least nine biological replicates (*n* ≥ 9). Different letters denote significant differences (*P* < 0.05, ANOVA and post hoc Tukey’s HSD test). Assessment of bacterial numbers in leaves 2 h after inoculation with *Psm lux* for the experimental settings of A–C is depicted in Supplemental Figure S1.

To further examine the roles of the SA pathway and the transcriptional coregulator NPR1 in NHP-triggered SAR, we pretreated the Col-0 wild-type, the SA-induction-deficient *sid2-1*, *sid2-2*, and *pbs3-1* mutants, as well as *npr1-3* with a 1 mM NHP solution via the soil and challenge-inoculated the leaves of the NHP-pretreated and water (H_2_O)-pretreated control plants with a compatible strain of the bacterial pathogen *Pseudomonas syringae* pv. *maculicola* (*Psm*) 1 d later. In the leaves of susceptible Arabidopsis, *Psm* is able to rapidly multiply in the apoplastic space and causes leaf chlorosis, while SAR-induced plants significantly prohibit bacterial growth and essentially prevent the development of disease symptoms (Gruner et al., 2018). Assessment of bacterial growth 2.5 d after the challenge inoculation with *Psm* revealed a strong NHP-induced SAR in the Col-0 wild-type (Figure 1A; Supplemental Figure S1A). As observed previously (Hartmann et al., 2018), NHP-triggered SAR was strongly diminished but not fully absent in the SA-induction-deficient *sid2* mutants. Moreover, *pbs3-1*, a mutant compromised in IC to SA conversion within the SA biosynthetic pathway (Rekhter et al., 2019; Torrens-Spence et al., 2019), behaved similarly than *sid2* (Figure 1A). In addition, upon soil treatment with NHP, the SA perception-defective *npr1-3* mutant only showed a weak and statistically not significant tendency of SAR activation toward leaf attack by *P. syringae* (Figure 1A). Further, when individual leaves of Col-0 plants were infiltrated with NHP, the same leaves developed strong acquired resistance to subsequent *Psm* infection (Figure 1B; Supplemental Figure S1B). In contrast, the treated leaves of *sid2-1* or *sid2-2* mutants only showed a modest resistance induction, consistent with the previously reported requirement of an intact SA pathway for strong Pip- and NHP-induced SAR (Bernsdorff et al., 2016; Hartmann et al., 2018). Moreover, *npr1-1* and *npr1-3* mutant lines completely failed to induce resistance in NHP-treated leaves, corroborating a requirement of functional NPR1 for NHP-triggered immunity (Figure 1B). Notably, when lower rosette leaves of Col-0 were treated with NHP, upper rosette leaves developed a strong SAR toward *P. syringae* infection (Figure 1C; Supplemental Figure S1C). This leaf-to-leaf transmitted resistance response only partly developed in the SA-deficient *sid2-2* line. This residual SAR response observed for *sid2* was absent in the *npr1-1* single mutant and in a *sid2-2 npr1-1* double mutant (Figure 1C). Together, this suggests that local elevation of NHP triggers a major SA-dependent and a weaker SA-independent branch of systemic, leaf-to-leaf immune signaling, and that both branches are transduced via NPR1.

**Figure 2:**
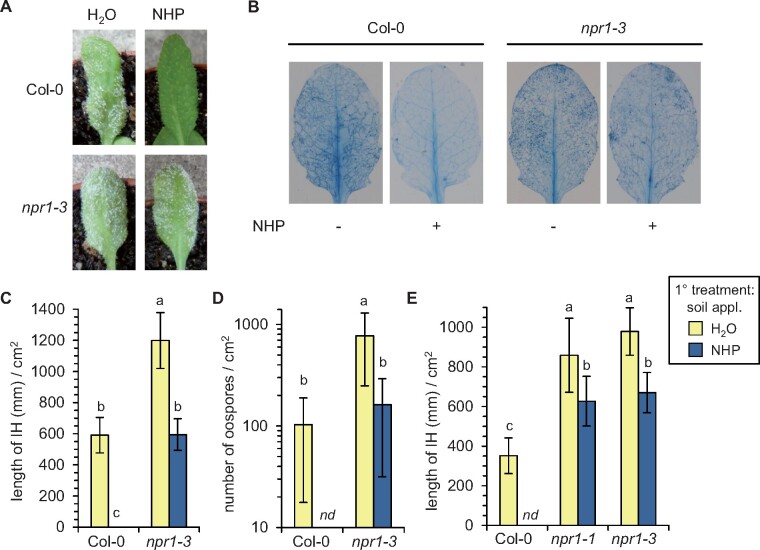
Exogenous NHP induces NPR1-dependent SAR against invasion by the biotrophic oomycete *Hpa*. The compatible isolate *Hpa* Noco2 invades Arabidopsis leaves via epidermal penetration and establishes IH within the leaf tissue. Subsequently, conidiophores bearing asexual conidiospores and spherical, sexual oospores are produced on the leaf surface, which is accompanied by the development of macroscopic downy mildew symptoms (Slusarenko and Schlaich, 2003). A, Individual Arabidopsis plants were supplied with 10 mL of an aqueous 1 mM NHP solution or with 10 mL of H_2_O via the soil. One day later, plants were spray-inoculated with a suspension of sporangia (5 × 10^4^ mL^−1^) of *Hpa* Noco2. Photographic images of leaves were taken 7 d after inoculation to illustrate representative downy mildew symptoms. The leaves of NHP-pretreated Col-0 plants were symptom-free throughout. B, Representative Trypan blue-stained leaves of *Hpa* Noco2-inoculated Arabidopsis plants pretreated with H_2_O (−) or NHP (+) are shown. Leaves were harvested and stained at 7 dpi. C and E, Quantitative assessment of the length of IH (mm)/cm^2^ leaf area in H_2_O- and NHP-pretreated plants at 7 dpi. The mean values (±sd) of at least 10 leaves from different plants are given (*n* ≥ 10). D, Number of oospores/cm^2^ leaf area at 7 dpi. The mean values (±sd) of at least nine leaves from different plants are given (*n* ≥ 9). Different letters denote significant differences (*P* < 0.05, ANOVA and post hoc Tukey’s HSD test). Nd, not detected.

Our previous results indicated that NHP systemically protects Arabidopsis from infection by the oomycete *Hyaloperonospora arabidopsidis* (*Hpa*; Hartmann et al., 2018). By employing *npr1* mutant plants in infection assays, we now assessed the role of NPR1 in NHP-induced resistance against the compatible *Hpa* isolate Noco2. In non-pretreated Col-0 plants, whitish downy mildew symptoms on leaves were readily observable at 7 d postinoculation (dpi) with *Hpa* (Figure 2A). At this infection stage, a dense network of intercellular hyphae (IH), which we visualized by Trypan blue staining (Figure 2B; Hartmann et al., 2018), developed in the leaf tissue, and numerous oospores were detected on the surfaces of leaves (Figure 2, C–E). Strikingly, the development of mildew symptoms, IH, and oospores was suppressed in the NHP-pretreated Col-0 plants, corroborating our previous findings that NHP-triggered SAR effectively prohibits the invasive growth of *Hpa* in the Col-0 leaf tissue (Figure 2; Hartmann et al., 2018). The mildew symptoms on non-pretreated *npr1* mutant plants at 7 dpi appeared more severe than those on wild-type plants (Figure 2A). Moreover, the lengths of IH/cm^2^ leaf surface were about twice as high as those of naïve Col-0 plants (Figure 2, C and E), and the *npr1* leaves also carried a higher number of oospores than Col-0 leaves at this stage of infection (Figure 2D). These results indicate that basal resistance to *Hpa* Noco2 is weaker in *npr1* plants than in the wild-type. Further, the disease symptoms developing in NHP pretreated *npr1* mutants were almost as severe as those of non-pretreated *npr1* upon *Hpa*-inoculation (Figure 2A). Although the extent of IH development and particularly of oospore formation was reduced significantly by NHP, a broad development of invasive hyphae and oospores was still discernible in NHP pretreated *npr1* plants (Figure 2). Therefore, the NHP-triggered acquired resistance that provided strong protection against *Hpa* invasion largely depended on a functional *NPR1* gene. Together, our resistance assays show that NPR1 is a central component of NHP-triggered SAR to bacterial and oomycete infection in Arabidopsis.

### The NHP-triggered transcriptional activation of SAR genes is strongly dependent on NPR1

Previous analyses suggested that NHP could activate certain sectors of defense-related gene expression. On the one hand, pathogen-induced SAR, which is triggered by the endogenous accumulation of NHP, is associated with a large transcriptional response systemically in the Arabidopsis foliage that includes upregulation of ˃3,000 genes (SAR^+^ genes) and downregulation of a similar number of genes (SAR^−^ genes; Bernsdorff et al., 2016). Significantly, this transcriptional SAR response fully depended on the function of the NHP biosynthetic genes *ALD1* and *FMO1* (Gruner et al, 2013; Bernsdorff et al., 2016). On the other hand, exogenous application of the NHP biosynthetic precursor Pip is sufficient to induce a significant subset of SAR^+^ genes in Arabidopsis leaf tissue, and this Pip-induced transcriptional response depended on a functional FMO1 monooxygenase, which catalyzes the N-hydroxylation of Pip to NHP (Hartmann et al., 2018).

**Figure 3:**
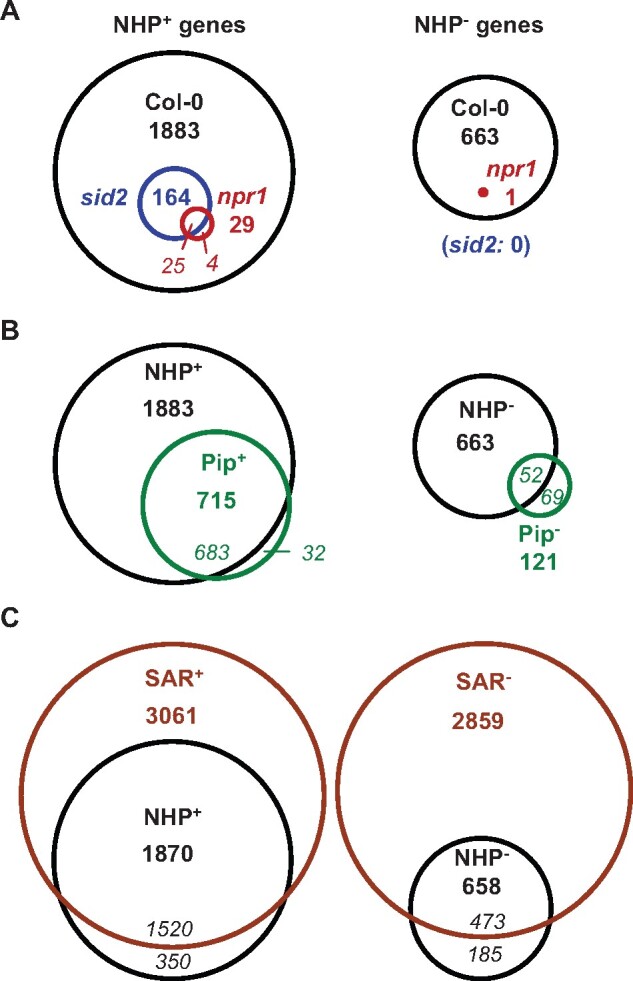
Transcriptional reprogramming of Arabidopsis upon exogenous NHP treatment widely overlaps with the transcriptional SAR response and depends on functional NPR1. Plants were watered with 10 mL 1 mM NHP (equal to doses of 10 µmol per plant), with 10 mL of 1 mM Pip, or with 10 mL of H_2_O as the control condition, and leaves were harvested one day later for RNA-seq analysis. The analysis was based on three independent leaf RNA samples per treatment and genotype that were obtained by conducting three separate experiments (Supplemental Tables S1 and S6). To determine statistically significant changes in gene expression of treatment versus control conditions and define genes up (NHP^+^/Pip^+^) and downregulated (NHP^−^/Pip^−^) by NHP/Pip, an FDR of *P* ≤ 0.05 was assumed (Benjamini and Hochberg, 1995). In addition, genes were only classified as NHP^+^ (Pip^+^) and NHP^−^ (Pip^−^) genes if the means of expression values of the treated samples related to those of the H_2_O-control samples exhibited a fold-change ˃1.5 and ˂0.67, respectively. A, Venn diagrams with numbers of differentially regulated genes out of 27,654 RNA-seq-covered genes between NHP- and H_2_O-treatments in the leaves of wild-type Col-0 (black), *sid2-1* (blue), and *npr1-3* (red) plants (bold numbers). The italicized numbers denote the number of overlapping and nonoverlapping genes between two genotypes (not given if overlap is complete). Left: upregulated (NHP^+^) genes. Right: downregulated (NHP^−^) genes. B, Venn diagrams with numbers of NHP-regulated (black) and Pip-regulated (green) genes in Col-0 (out of 27,654 total genes). Left: upregulated (NHP^+^/Pip^+^) genes. Right: downregulated (NHP^−^/Pip^−^) genes. Italicized numbers denote the number of overlapping and nonoverlapping NHP^+^ and Pip^+^ (NHP^−^ and Pip^−^) genes. C, Venn diagrams with numbers of NHP-regulated genes (black) and genes differentially regulated in biologically induced SAR (brown). The SAR^+^ (SAR^−^) genes constitute upregulated (downregulated) genes in upper leaves of Col-0 plants inoculated 2 d earlier in lower leaves with *Psm*, as compared to a mock-treatment (FDR < 0.05; *n* = 3; Bernsdorff et al., 2016; Hartmann et al., 2018). Only genes present in both the NHP- and SAR-gene datasets (26,711 in total) were considered in (C). Italicized numbers denote the number of overlapping and nonoverlapping NHP^+^ and SAR^+^ (NHP^−^ and SAR^−^) genes.

To directly test whether elevation of NHP levels is sufficient to induce SAR-associated transcriptional reprogramming, we supplied Arabidopsis plants with NHP exogenously by the soil treatment mode that was previously applied to investigate the Pip-inducible transcriptional response (Hartmann et al., 2018). Individual plants were watered with 10 mL of 1-mM NHP solution or with 10 mL of H_2_O, and leaves were harvested 1 d later for transcriptional analysis at the whole genome level by RNA-sequencing (RNA-seq; Supplemental Table S1). To assess possible SA- and NPR1-dependencies of NHP-induced gene expression, we employed, besides the Col-0 wild-type, the *sid2-1* and *npr1-3* mutants in this study. To directly compare the wild-type response to NHP with the response to its biosynthetic precursor Pip, we also included the analogous Pip treatment (watering with 10 mL of 1-mM Pip) for Col-0 in each of the three independent experiments (Figure 3; Supplemental Table S1). We identified 1,883 “NHP^+^” and 663 “NHP^−^” genes out of 27,654 totally RNA-seq-covered genes that were significantly up and downregulated in the Col-0 wild-type by the NHP treatment, respectively (Figure 3A). The response to Pip in the wild-type (715 “Pip^+^” and 121 “Pip^−^” genes) was qualitatively similar but quantitatively smaller than the response to NHP (Figure 3B; Supplemental Figure S2). On the one hand, strong overlap between NHP- and Pip-regulated genes existed, as exemplified by the fact that 683 (i.e. 96%) of the 715 Pip^+^ genes were also NHP^+^ genes (Figure 3B). On the other hand, many of the 1,200 genes classified as NHP^+^ but not as Pip^+^ genes were tendentially also upregulated by Pip, but the lower response and the variation between experiments resulted in false discovery rate (FDR) values ˃0.05 for the Pip-treatment so that these genes were finally not classified as Pip^+^ genes (Supplemental Figure S2).

By a similar RNA-seq approach, we previously examined the transcriptional changes in the upper leaves of Arabidopsis plants in response to a previous inoculation of lower leaves with *Psm.* Out of a total of 28,496 covered genes, we identified 3,230 SAR^+^ genes upregulated and 3,018 SAR^−^ genes downregulated during this biological SAR response (Bernsdorff et al., 2016). We merged the present and previous RNA-seq datasets on NHP- and SAR-regulated gene expression, which yielded a total list of 26,711 genes present in both datasets (Figure 3C). Out of these, 3,061 (2,859) genes were SAR^+^ (SAR^−^) genes and 1,870 (658) genes were NHP^+^ (NHP^−^) genes. Notably, 1,520 (i.e. 81%) of the NHP^+^ genes also represented SAR^+^ genes, whereas 473 (i.e. 72%) of the NHP^−^ genes were SAR^−^ genes (Figure 3C). Therefore, the transcriptional responses of Arabidopsis Col-0 to exogenous NHP and biological SAR induction largely overlap.

We next investigated whether the NHP^+^ and NHP^−^ genes were enriched or depleted in particular gene ontology (GO) categories by using The Arabidopsis Information Resource (TAIR) GO Term Enrichment Tool (https://www.arabidopsis.org/tools/go_term_enrichment.jsp; Bernsdorff et al., 2016; Hartmann et al., 2018). Many GO categories were significantly overrepresented among the NHP^+^ genes, for example, the terms “response to biotic stimulus,” “defense response,” “systemic acquired resistance,” “signal transduction,” “cell surface receptor signaling,” “calcium/calmodulin binding,” “protein cell death,” and “response to ER stress” (Table 1; Supplemental Table S2). A few categories, including “photosynthetic membrane” or “gene expression” were also underrepresented in the NHP^+^ gene group. In addition, based on the merged gene set, we analyzed whether particular Arabidopsis gene families would be enriched or depleted in the different groups of NHP- and SAR-regulated genes. We found that RLKs, RLPs, resistance proteins, mitogen-activated protein (MAP) kinases (MAPKs), calcium (Ca^2+^)-dependent protein kinases (CDPKs), and WRKY transcription factors belonged to the gene families over-proportionally upregulated by NHP. The GO categories and gene families over-represented in the NHP^+^ group were generally also enriched in the group of SAR^+^ genes (Table 1; Supplemental Table S2).

**Table 1:** Occurrence of NHP^+^ and SAR^+^ genes in groups of GO terms and gene families

	Gene Category	Number of Genes in			% Gene Category in			Fold-Enrichment	
		Genome	NHP^+^	SAR^+^	Genome	NHP^+^	SAR^+^	NHP^+^	SAR^+^
A	GO Term Analyses								
	Total Number of Genes	27,416	1,854	3,027	–	–	–	–	–
	Response to Biotic Stimulus	1,022	255	581	3.7	13.8	11.9	3.7^***^	3.2^***^
	Defense Response	1,005	239	327	3.7	12.9	10.8	3.5^***^	3.0^***^
	SAR	61	26	26	0.2	1.4	0.9	6.3^***^	3.9^***^
	Response to SA	206	63	77	0.8	3.4	2.5	4.5^***^	3.4^***^
	Signal Transduction	1,300	191	291	4.7	10.3	9.6	2.2^***^	2.0^***^
	Cell Surface Receptor Signaling	54	19	17	0.2	1.0	0.6	5.9^***^	2.9
	Protein Phosphorylation	799	142	192	2.9	7.7	6.3	2.6^***^	2.2^***^
	Ca^2+^ Ion Binding	199	47	63	0.7	2.5	2.1	3.5^***^	2.9^***^
	Calmodulin Binding	146	34	55	0.5	1.8	1.8	3.4^***^	3.4^***^
	Response to ROS	151	38	46	0.6	2.1	1.5	3.7^***^	2.8^***^
	Cell Death	112	37	46	0.4	2.0	1.5	4.9^***^	3.7^***^
	Response to ER Stress	97	37	55	0.4	2.0	1.8	5.6^***^	5.1^***^
	Protein Folding	169	36	37	0.6	1.9	1.2	3.2^***^	2.0
	Photosynthetic Membrane	424	2	13	1.6	0.1	0.4	0.1^***^	0.3^***^
	Ribosome Structural Constituent	281	1	4	1.0	0.1	0.1	0.1^***^	0.1^***^
	Gene Expression	1,403	48	85	5.1	2.6	2.8	0.5^***^	0.5^***^
B	Family Analyses								
	Total Number of Genes	26,711	1,870	3,061	–	–	–	–	–
	RLK	577	141	157	2.16	7.54	5.13	3.5^***^	2.4^***^
	CRK	41	25	29	0.15	1.34	0.95	8.7^***^	6.2^***^
	RLP	54	28	21	0.20	1.50	0.69	7.4^***^	3.4^***^
	R Proteins (NBS-LRR)	166	55	62	0.62	2.94	2.03	4.7^***^	3.3^***^
	MAPK(K)(K)	88	20	30	0.33	1.07	0.98	3.2^***^	3.0^***^
	CDPK	34	9	13	0.13	0.48	0.42	3.8^**^	3.3^***^
	CNGC	20	6	7	0.07	0.32	0.23	4.3^**^	3.1^*^
	WRKY	71	23	27	0.27	1.23	0.88	4.6^***^	3.3^***^
	NAC	107	18	31	0.40	0.96	1.01	2.4^**^	2.5^***^
	CHI	24	6	6	0.09	0.32	0.20	3.6^*^	2.2
	HSP	57	14	14	0.21	0.75	0.46	3.5^***^	2.1^*^
	DOX	129	16	23	0.48	0.86	0.75	1.8^*^	1.6
	LCR	84	0	0	0.31	0.00	0.00	0.0^**^	0.0^***^

A, NHP^+^ and SAR^+^ genes in distinct GO term categories (https://www.arabidopsis.org/tools/go_term_enrichment.jsp). The first row depicts the total number of genes in the reference list of the TAIR gene enrichment tool and the numbers of NHP^+^ and SAR^+^ genes in this list (see also Figure 3). The other rows depict the absolute number of genes of a particular GO category in the whole genome, in the NHP^+^ group and in the SAR^+^ group (left columns), the percentages of genes from the GO categories in the whole genome, NHP^+^ and SAR^+^ groups (middle columns), and the fold-enrichment of the NHP^+^ and SAR^+^ gene groups with respect to the whole genome (asterisks indicate significant enrichment or depletion; Fisher’s exact test.

* *P* < 0.05,

** *P* < 0.001,

*** *P* < 0.0001.

B, Gene family analysis based on TAIR10 family annotation and published lists of gene families. The total genes used for family analysis (26,711) comprised the merged list of total genes covered in both the NHP- and SAR-related RNA-seq analyses (Figure 3C). See (A) for further information. MAPK(K)(K), MAPK cascade members; CNGC, cyclic nucleotide-gated ion channels; WRKY, WRKY-domain transcription factors; NAC, NAM/ATAF/CUC transcription factor family; CHI, chitinases; HSP, heat shock proteins; DOX, 2-oxoglutarate-dependent dioxygenases; LCR, low molecular weight, cysteine-rich (defensin-like proteins). See also Supplemental Table S2.

**Figure 4:**
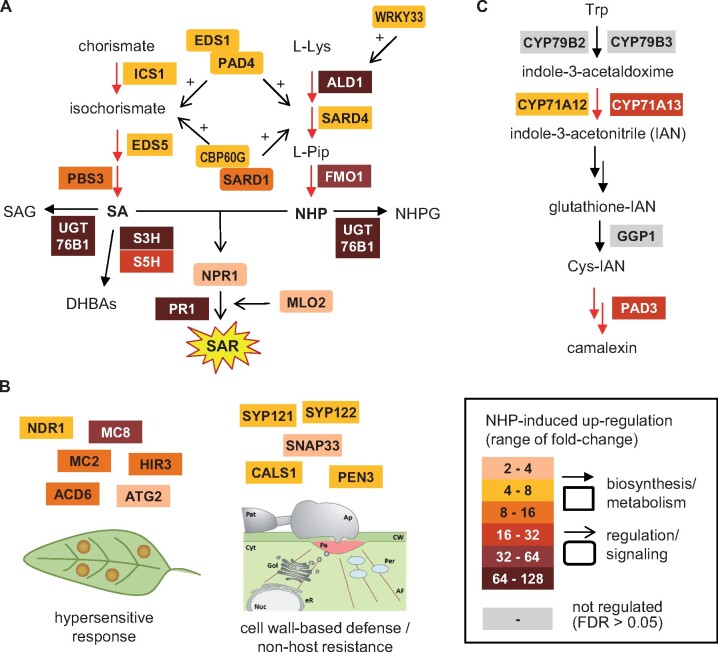
NHP upregulates a battery of plant defense-related genes involved in distinct immune layers. Selected expression data of the RNA-seq analysis is illustrated. A, Genes involved in the biosynthesis and metabolism of SA and NHP, and in the regulation of the respective immune pathways. B, Genes implicated in hypersensitive cell death, cell-wall-based defense, and nonhost resistance. C, Genes involved in the biosynthesis of the Arabidopsis phytoalexin camalexin (Mucha et al., 2019). Genes significantly upregulated in the Col-0 wild-type upon NHP treatment (FDR < 0.05) are highlighted in color, genes not differentially regulated are depicted in grey. The heat map indicates the fold-changes of gene transcript levels (i.e. the ratios of the mean expression values of NHP versus control samples). Please note that all of the SA and NHP biosynthetic genes are invariably induced by NHP at the transcriptional level, whereas camalexin biosynthesis is only partially activated.

More detailed analyses at the individual gene level revealed that all of the genes involved in the biosynthesis of SA and NHP as well as in the regulation of these two immune pathways were significantly upregulated by NHP (Figure 4A; Supplemental Table S3). Moreover, several genes that have been functionally associated with the execution of hypersensitive cell death, cell wall-based defense, and nonhost resistance belonged to the NHP-inducible genes (Figure 4B; Supplemental Table S3). In addition, NHP partially activated the biosynthesis of the Arabidopsis phytoalexin camalexin, since three out of the six characterized camalexin biosynthetic genes belonged to the NHP^+^ gene group (Figure 4C; Supplemental Table S3; Mucha et al., 2019). Together, these results suggest that NHP activates molecular components involved in distinct layers and signaling stages of the plant immune system.

Whereas induction of SAR by pathogen inoculation led to up and downregulation of a similar number of genes (Bernsdorff et al., 2016), the majority of the differentially expressed genes were up rather than downregulated in response to the NHP treatment (Figure 3C). The previously characterized SAR^−^ response is associated with a decreased expression of a high proportion of photosynthesis-related genes (Bernsdorff et al., 2016). For example, out of 177 genes (0.7% of the total number of genes) annotated to the GO term “photosynthesis,” 113 genes (i.e. 6.2% of the SAR^−^ genes) belonged to the SAR^−^ group (Table 2A). Notably, the prominent downregulation of genes from photosynthesis- and chloroplast-related categories detected during the pathogen-induced SAR response was not observed to the same extent in the NHP response (Table 2A; Supplemental Table S4). Another previously described hallmark of the SAR^−^ group was a significant enrichment in genes coding for fasciclin-like arabinogalactan proteins (FLAs), expansins (EXP), and xyloglucan endotransglucosylase/hydrolases (XTH; Bernsdorff et al., 2016). In this case, a similar trend was observed for the NHP^−^ gene group (Table 2B). Together, this indicates that gene downregulation is triggered in a qualitatively similar manner following biological SAR induction and exogenous NHP treatment but that it is quantitatively more pronounced during the biological SAR response.

**Table 2:** Occurrence of NHP and SAR genes in groups of GO terms and gene families

	Gene Category	Number of Genes in			% GO Term Genes in			Fold-Enrichment	
		Genome	NHP^−^	SAR^−^	Genome	NHP^−^	SAR^−^	NHP^−^	SAR^−^
A	GO Term Analyses								
	Total Number of Genes	27,416	657	2,832	–	–	–	–	–
	Photosynthesis	177	10	113	0.7	1.5	4.0	2.4	6.2^***^
	Phot. Electron Transport Chain	40	4	30	0.2	0.6	1.1	4.2	7.3^***^
	Red. Pentose Phosphate Cycle	13	0	11	0.05	0.0	0.4	0.0	8.2^*^
	Chloroplast	5,059	178	1,255	18.5	27.1	44.3	1.5^***^	2.4^***^
	Chl. Thylakoid Membrane	406	17	251	1.5	2.6	8.9	1.8	6.0^***^
	Chl. Stroma	767	32	399	2.8	4.9	14.1	1.7	5.0^***^
	Chl. Envelope	670	32	348	2.4	4.9	12.3	2.0	5.0^***^
	Chlorophyll Biosynthesis	37	4	21	0.1	0.6	0.7	4.5	5.5^***^
	Carotenoid Metabolism	30	3	16	5.7	11.8	11.1	4.2	5.2^**^
	Apoplast	431	37	119	1.6	5.6	4.2	3.6^***^	2.7^***^
	Membrane	7,514	240	1,137	27.41	36.53	40.15	1.3^***^	1.5^***^
	H_2_O Transport	29	9	13	0.1	1.4	0.5	13.0^***^	4.3
	Organelle Lumen	1,304	7	69	4.8	1.1	2.4	0.2^***^	0.5^***^
	Nucleus	10,597	204	854	38.7	31.1	30.2	0.8^*^	0.8^***^
	Nucleic Acid Metabolism	1,629	8	140	4.8	1.1	2.4	0.2^***^	0.8
B	Family Analyses								
	Total Number of Genes^b^	26,711	658	2,858	–	–	–	–	–
	FLA	25	4	11	0.09	0.61	0.38	6.5^**^	4.1^***^
	XTH	33	5	9	0.12	0.76	0.31	6.2^**^	2.5^*^
	EXP	35	2	10	0.13	0.30	0.35	2.3	2.7^**^
	PIP	13	8	10	0.05	1.22	0.35	25.0^***^	7.2^***^

A, NHP and SAR genes in distinct GO term categories.

B, Gene family analysis. See Table 1 for further details. Asterisks indicate significant enrichment or depletion; Fisher’s exact test; *P < 0.05, **P < 0.001, ***P < 0.0001.

FLA: fasciclin-like arabinogalactan proteins; XTH: xyloglucan endotransglucosylase/hydrolases; EXP: expansins; PIP, plasma membrane intrinsic proteins. See also Supplemental Table S3.

We next compared the genes that were differentially regulated upon NHP treatment in the Col-0, *sid2-1*, and *npr1-3* plants. Compared to the 1,883 NHP^+^ genes upregulated in the Col-0 wild-type, only 8.7% (164 genes) were upregulated in *sid2-1*, and as few as 29 genes (1.5%) were induced in *npr1-3*. All the genes upregulated in *sid2-1* and *npr1-3* fell into the NHP^+^ gene cluster defined for Col-0 (Figure 3A). Out of the 29 genes that were induced by NHP in *npr1-3*, 25 were also induced in *sid2-1*. In addition, the NHP^−^-response that was readily discernable in the Col-0 wild-type was virtually absent in both *sid2-1* and *npr1-3* mutants (Figure 3A). Together, this indicates that the transcriptional response to NHP is largely dependent on the capability of plants to induce the biosynthesis of SA. To an even greater extent, gene induction by NHP depends on the function of the transcriptional coactivator NPR1.

**Figure 5:**
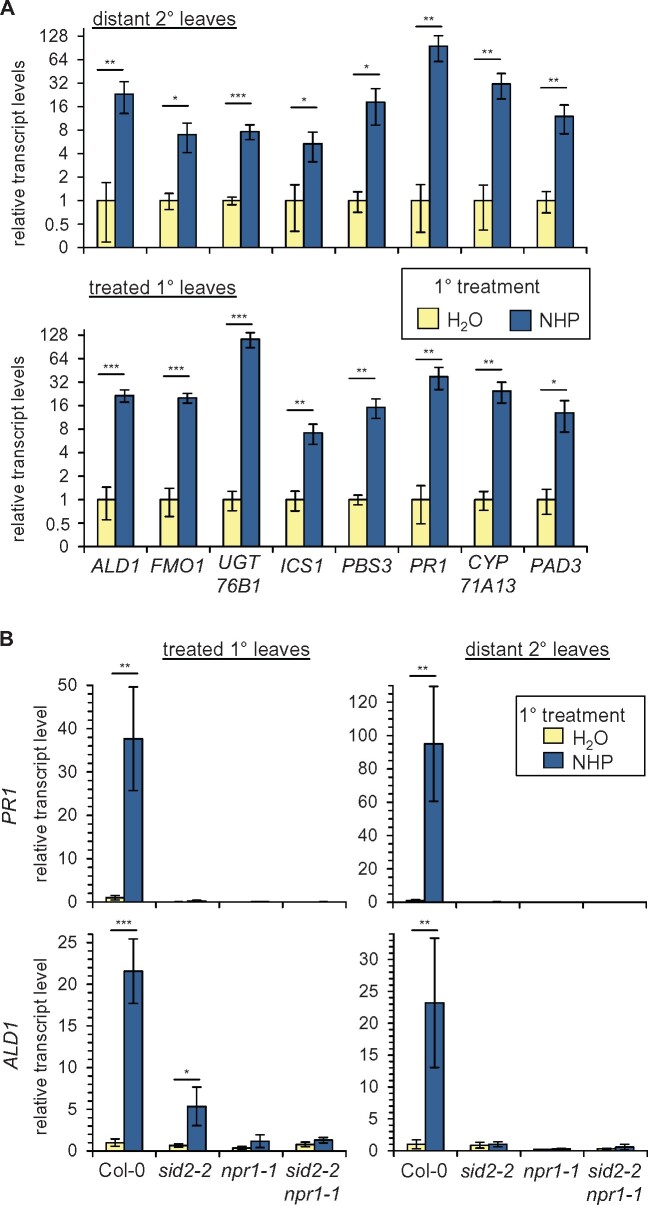
Local and systemic upregulation of immune-related genes involved in the metabolism or signaling pathways of NHP, SA, and camalexin upon treatment of Arabidopsis leaves with NHP. A, Relative transcript levels of indicated genes in leaves of Col-0 plants infiltrated with 1 mM NHP or H_2_O (treated 1° leaves, lower graph), and in distant nontreated 2° leaves (upper graph) at 24-h post-1° leaf treatment, as assessed by RT-qPCR analysis. B, Relative transcript levels of *PR1* and *ALD1* in treated (1°) leaves of Col-0, *sid2-2*, *npr1-1*, and *sid2-2 npr1-1* plants infiltrated with NHP or H_2_O (left graphs), and in untreated, distant (2°) leaves of the same plants (right graphs). Transcript levels of genes are given as means ± sd of four biological replicates (*n* = 4) and are expressed relative to the respective H_2_O-control value. Asterisks denote statistically significant differences between NHP- and H_2_O-treatments (*P < 0.05, **P < 0.01, ***P < 0.001; two-tailed *t* test).

As outlined above, a local treatment of NHP induced acquired resistance to subsequent infection in both the treated leaves and in leaves distant from the initial NHP application (Figure 1, B and C). To investigate whether selected NHP^+^ genes that were significantly induced by the soil treatment mode would also be upregulated by leaf treatment, we assessed expression of SA- (*ICS1, PBS3*, and *PR1*), NHP- (*ALD1, FMO1*, and *UGT76B1*), and camalexin-(*CYP71A13, PHYTOALEXIN-DEFICIENT3* [*PAD3*])-related genes upon infiltration of lower leaves in the treated (local response) or in distant, upper (systemic response) leaves of the same plants by reverse transcription-quantitative PCR (RT-qPCR)-based analyses. Notably, leaf treatment with NHP resulted in a strong upregulation of each of the tested genes in both the local and the distant leaf tissue, indicating a systemic action of NHP on defense-related gene expression (Figure 5A). We then examined the transcript levels of two of these genes, *ALD1* and *PR1*, for which we had previously established distinct SAR-related activation modes (Bernsdorff et al., 2016), in *sid2-2*, *npr1-1*, and *sid2-2 npr1-1* plants (Figure 5B). The *PR1* gene represents a classical marker for SA-activated defense signaling, and *PR1* was upregulated in the distal leaves of locally pathogen-inoculated plants in dependence of the SA biosynthetic gene *ICS1/SID2* (Bernsdorff et al., 2016). We found that NHP-induced increases in *PR1* transcript levels in local and systemic leaf tissue fully depended on functional *SID2* and *NPR1* genes (Figure 5B). *ALD1* was also classified as a strongly upregulated SAR gene in our previous study, but the pathogen-induced expression of this gene in distal leaf tissue occurred in a partial SA-independent manner (Bernsdorff et al., 2016). In contrast to *PR1*, a diminished but still significant increase of *ALD1* transcript levels was observed in NHP-treated *sid2-2* leaves, whereas the local induction of *ALD1* expression was entirely absent in *npr1-1* and *sid2-2 npr1-1* plants. Moreover, *ALD1* transcript levels were elevated in the distant leaves of neither *sid2-2*, *npr1-1*, nor *sid2-2 npr1-1* plants upon local NHP application (Figure 5B). Therefore, the induction of *PR1* and *ALD1* expression by NHP in both local and systemic leaves required NPR1. Remarkably, the NHP-induced local elevation of *ALD1* transcript levels was only partially dependent on SA biosynthesis.

### Movement of NHP from treated to distant leaves occurs independently of a functional SA pathway

**Figure 6:**
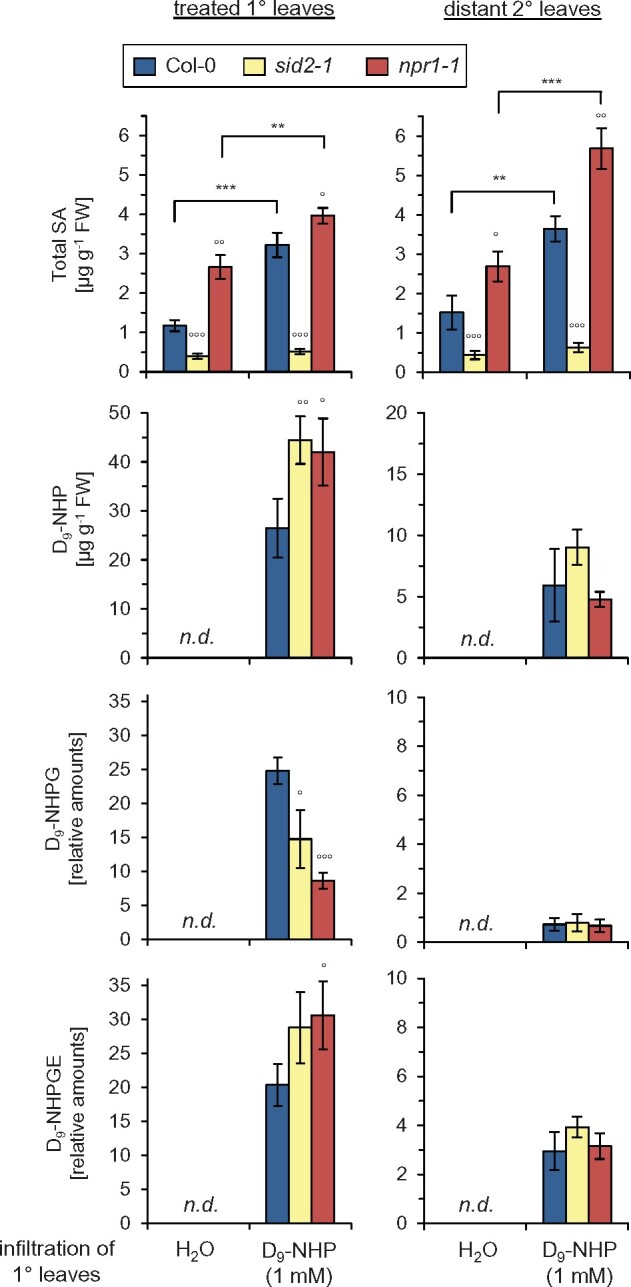
Leaf-applied NHP translocates from treated to distant leaves, are partially glycosylated, and induces systemic SA accumulation in an *NPR1*-independent manner. Lower, 1° leaves of Arabidopsis Col-0, *sid2-1*, or *npr1-1* were infiltrated with 1 mM deuterium-labeled NHP (D_9_-NHP), and the contents of D_9_-NHP, D_9_-NHPG, D_9_-NHPGE, SA, SAG, and SGE were determined 24 h later in the 1° treated and in distant upper (2°) leaves. Total SA represents the sum of unconjugated SA and the two glycosylated forms (Supplemental Figure S3 individually depicts accumulation of free SA, SAG, and SGE). SA and D_9_-NHP levels are given in µg g^−1^ FW. Due to the unavailability of authentic standards, the levels of D_9_-NHPG and D_9_-NHPGE are given as relative, FW-related amounts. Bars represent means ± sd of four biological replicates (*n* = 4). Asterisks indicate statistically significant differences between the H_2_O-control and the D_9_-NHP treatments for a particular genotype (^***^P < 0.001, ^**^P < 0.01; two-tailed *t* test). Circles denote statistically significant differences of Col-0 and mutant samples within a same treatment (°°°*P* < 0.001, °°*P* < 0.01, °*P* < 0.05; two-tailed *t* test). Similar results were obtained when plants were treated with 1 mM NHP instead of 1 mM D_9_-NHP (Supplemental Figure S4).

We next asked whether a localized leaf application of NHP would be sufficient to activate SA biosynthesis systemically in the Arabidopsis foliage. As it was proposed in previous studies that exogenously applied NHP might move from the treated to distant leaf tissue (Chen et al., 2018), we examined whether a possible leaf-to-leaf movement of NHP would require an intact SA biosynthetic pathway and/or NPR1. To allow unequivocal discrimination between exogenously applied and endogenously produced NHP, we employed, besides NHP, the deuterated variant D_9_-NHP in these experiments. Infiltration of lower leaves of Col-0 plants with solutions of either 1 mM D_9_-NHP or 1 mM NHP induced the accumulation of unconjugated SA, the SA-β-glucoside (SAG), and the SA glucose ester (SGE) in both the treated and in distant leaves at 24 h after the treatment (Figure 6; Supplemental Figures S3 and S4). Notably, *npr1-1*, which already exhibited elevated basal levels of total SA, also showed (D_9_)-NHP-induced systemic accumulation of these three forms of SA. Due to their defect in inducible SA biosynthesis, *sid2-1* plants failed to accumulate SA upon (D_9_)-NHP-treatment (Figure 6; Supplemental Figures S3 and S4).

In leaves treated with 1-mM D_9_-NHP solution, ∼30 µg g^−1^ of D_9_-NHP was detectable at 24 h after application (Figure 6). This is similar to the amount of NHP that accumulates endogenously in *Psm*-inoculated Arabidopsis plants in the course of SAR establishment (Hartmann et al., 2018). Notably, the untreated distant leaves also contained ∼5 µg g^−1^ of D_9_-NHP upon the local treatment, indicating that a substantial amount of D_9_-NHP was translocated from the treated to the distant leaves (Figure 6). This leaf-to-leaf movement of D_9_-NHP proved independent of SA signaling and NPR1, as *sid2-1* and *npr1-1* accumulated wild-type-like levels of D_9_-NHP in their distant leaves. In addition, the two recently characterized NHP glucosides, NHPG and NHPGE (Hartmann and Zeier, 2018; Bauer et al., 2021), were detected as deuterated variants in both the D_9_-NHP-treated und the distant Col-0 leaves (Figure 6). Interestingly, the level of unconjugated D_9_-NHP was lower in the treated leaves of Col-0 than in *sid2-1* and *npr1-1*, whereas the level of the D_9_-NHPG was higher in the treated wild-type leaves than in the mutants (Figure 6). This observation is in line with our recent finding that the conversion of NHP to NHPG by the UGT76B1 glycosyltransferase is promoted by an intact SA pathway (Bauer et al., 2021). The metabolic situation just described for the D_9_-NHP application was analogously observed for the NHP treatment (Figure 6; Supplemental Figure S4). Together, these results indicate that the leaf-to-leaf movement of exogenously applied NHP requires neither intact SA biosynthesis nor NPR1. Moreover, NHP is able to induce SA accumulation in both the local and the distant leaf tissue in an NPR1-independent manner.

**Figure 7:**
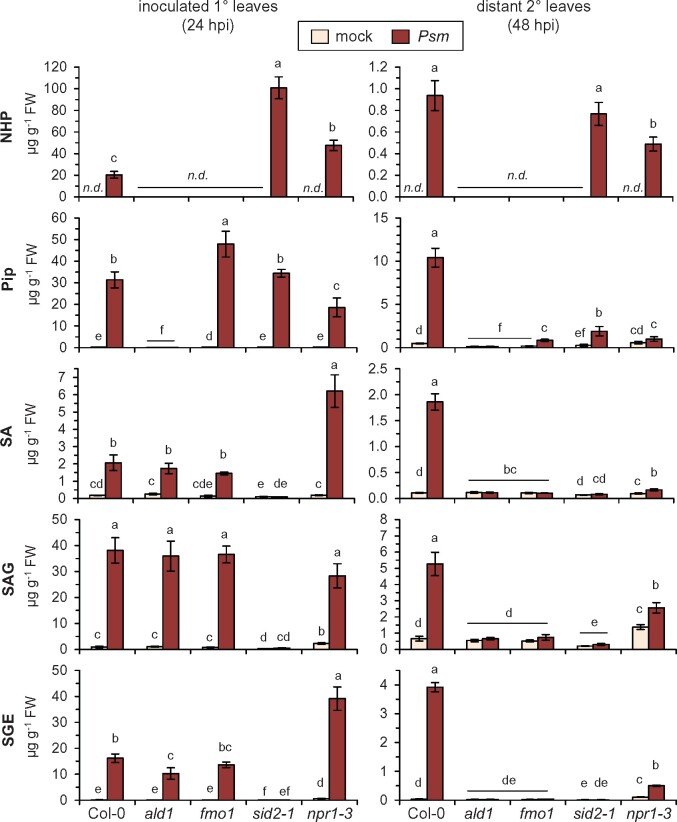
Accumulation of defense metabolites during biologically-induced SAR: NHP and SA derivatives increase systemically in *npr1* mutant plants. Levels of NHP, Pip, SA, SAG, and SGE in Arabidopsis Col-0 (wild-type), *ald1*, *fmo1*, *sid2-1*, and *npr1-3* plants inoculated with *Psm*-inoculated or mock-treated with 10 mM MgCl_2_. Left: local accumulation; metabolite levels (in µg g^−1^ FW) in lower, inoculated (1°) leaves at 24 hpi. Right: systemic accumulation; metabolite levels in upper, distant (2°) leaves at 48 after inoculation of lower leaves. Bars represent means ± sd of four biological replicates. Different letters denote significant differences (*P* < 0.05, Kruskal–Wallis *H* test).

To bring these findings into context with the biological SAR process, we examined the metabolic changes in *Psm*-inoculated lower and distant upper leaves of Arabidopsis Col-0, *ald1*, *fmo1*, *sid2-1*, and *npr1-3* plants (Figure 7). As reported previously (Bernsdorff et al., 2016; Hartmann et al., 2018), *ald1* was unable to accumulate Pip and NHP in response to *Psm*, *fmo1* lacked NHP accumulation, *sid2-1* failed to accumulate SA and its glucose conjugates SAG and SGE, and *sid2-1* and *npr1-3* both over-accumulated NHP in the locally inoculated leaves. Moreover, *ald1* and *fmo1* accumulated SA and its derivatives in a manner similar to the wild-type in the inoculated leaves. In contrast, a *Psm*-triggered over-accumulation of SA and SGE was observed in the local leaves of *npr1-3*, whereas accumulation SAG tended to be reduced (Figure 7). This suggests an involvement of NPR1 in the regulation of SA glycosylation.

SA and its derivatives did not accumulate in the distant leaves of *ald1* and *fmo1* in response to *Psm* attack, corroborating the requirement of NHP biosynthesis for systemic SA accumulation (Mishina and Zeier, 2006; Návarová et al., 2012; Hartmann et al., 2018). In contrast, both *sid2-1* and *npr1-3* markedly accumulated NHP in their distant leaves upon *Psm* inoculation (Figure 7). NHP shows leaf-to-leaf mobility in *sid2-1* and *npr1-3*, but SAR-related gene expression is strongly attenuated in these lines (Figures 3A and 6; Bernsdorff et al., 2016). Thus, the *Psm*-induced systemic increase of NHP in these lines might be a consequence of translocation from the inoculated to the distant leaves rather than de novo synthesis of NHP in distant leaves. The *npr1-3* mutant was in large part compromised in the systemic accumulation of free SA (Figure 7; Attaran et al., 2009). However, in particular SAG and SGE accumulated to some extent in the distant leaves of *npr1-3* in response to *Psm* attack (Figure 7). Therefore, the biosynthesis of SA in systemic tissue appears to be inducible to some degree independently of NPR1, as was also observed in the (D_9_)-NHP feeding experiments (Figure 6; Supplemental Figures S3 and S4).

### NHP primes plants for an effective activation of metabolic immune responses after pathogen attack

Plants can acquire an alarmed, primed state upon biotic or abiotic stress exposure which enables them to more successfully handle future stress situations (Hilker et al., 2016; Wilkinson et al., 2019). Our previous studies have shown that a localized leaf inoculation with SAR-inducing pathogens systemically primes the foliage of Arabidopsis to react more quickly toward subsequent pathogen challenge, and that this biological induction of priming fully requires the endogenous biosynthesis of NHP (Návarová et al., 2012; Bernsdorff et al., 2016; Hartmann et al., 2018). The pathogen-inducible plant responses primed in this manner included the accumulation of camalexin, Pip, and SA, as well as the expression of defense-related genes, such as *PR1*, *ALD1*, or *FMO1*. Moreover, plants were similarly primed for enhanced pathogen-induction of these immune responses if they were exogenously pretreated with the NHP biosynthetic precursor Pip. This Pip-induced priming was fully dependent on functional *FMO1*. On this basis, we previously concluded that NHP would act as the active priming inducer in SAR (Hartmann and Zeier, 2018).

**Figure 8:**
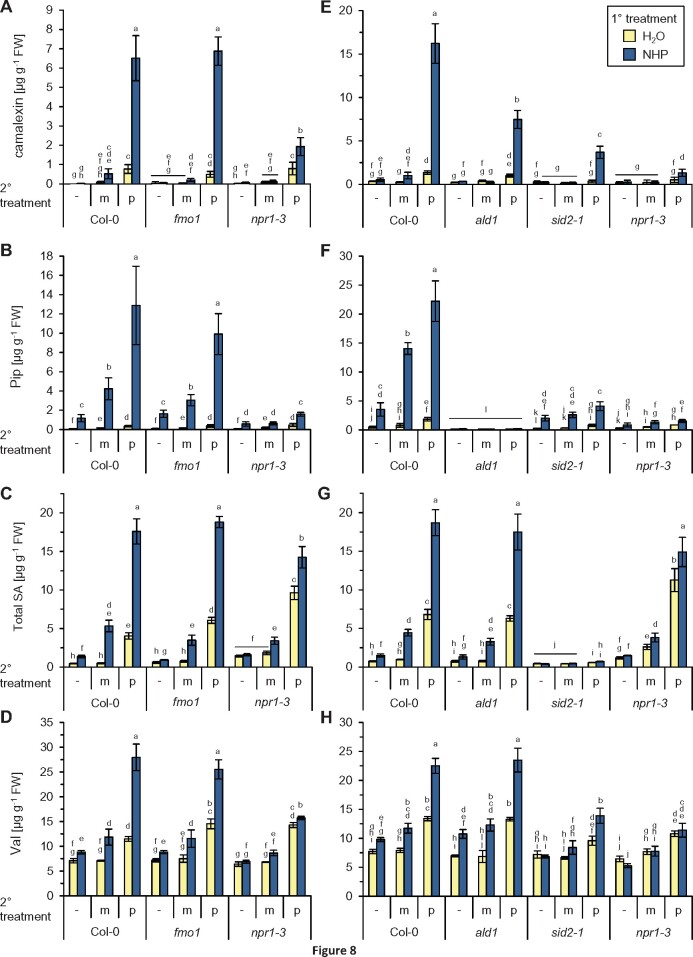
Exogenous NHP primes plants for enhanced pathogen-induced (defense) metabolite accumulation. A and E, Camalexin accumulation. B and F, Pip accumulation. C and G, Accumulation of total SA, that is, the sum of unconjugated SA, SA-SAG, and SGE. Accumulation of each of the three SA forms is primed by NHP (Supplemental Figure S5). D and H, Val accumulation. Accumulation of the other BCAAs, Leu and Ile, is also primed by NHP (Supplemental Figure S6). Plants were watered with 10 mL of 1 mM NHP or 10 mL of H_2_O (1° treatment), and leaves challenge-inoculated with *Psm* (p) or mock-infiltrated (m) with 10 mM MgCl_2_ 1 d later (2° treatment). The leaves of a third set of plants were left untreated (−). Metabolite levels in leaves were determined 12 h after the 2° treatment. Bars represent means ± sd of four biological replicates (*n* = 4). Different letters denote significant differences (*P* < 0.05, Kruskal–Wallis *H* test). A–D, Experiment with Col-0, *fmo1*, and *npr1-3*. E–H, Independent experiment with Col-0, *ald1*, *sid2-1*, and *npr1-3*.

To directly test whether elevated levels of NHP were sufficient to induce defense priming, we supplied plants with 1 mM NHP or with H_2_O as a control pretreatment according to the protocol previously employed to study Pip-inducible priming (Bernsdorff et al., 2016). One day later, we challenge-inoculated the leaves of a first subgroup of plants by infiltrating a suspension of *Psm*, leaf-infiltrated a second plant set with a mock-solution (10 mM MgCl_2_), or left the leaves of a third set of plants untreated. The leaves were then harvested 12 h later to assess the early induction of metabolic responses (Figure 8; Supplemental Figures S5 and S6). In the Col-0 wild-type, *Psm* inoculation of H_2_O pretreated control plants triggered a moderate accumulation of camalexin to ∼0.8–1.4 µg g^−1^ fresh weight (FW) at 12-h post-inoculation (hpi; Figure 8, A and E). Whereas NHP pretreatment alone was not sufficient to activate camalexin biosynthesis, the NHP pretreated plants accumulated ∼10-fold higher amounts of camalexin at 12-h post-*Psm* challenge (between 6.5 and 16 µg g^−1^ FW) than the H_2_O-pretreated plants (Figure 8, A and E), indicating a strong NHP-mediated priming of the pathogen-triggered accumulation of camalexin. This was reminiscent to the observed priming of camalexin biosynthesis in Col-0 plants pretreated with exogenous Pip or conditioned by a SAR-inducing pathogen inoculation (Návarová et al., 2012; Bernsdorff et al., 2016). Exogenous NHP also strongly primed the NHP-deficient *fmo1* mutant for the *Psm*-triggered accumulation of camalexin (Figure 8A). Thus, in contrast to exogenous Pip (Bernsdorff et al., 2016), exogenous NHP was able to restore the priming of camalexin accumulation in *fmo1*. NHP also partially restored camalexin priming in *ald1*, which is deficient in both Pip and NHP biosynthesis (Figure 8E). To test whether the NHP-inducible priming response requires an intact SA signaling pathway, we included the *sid2-1* and *npr1-3* mutants in the priming assay. We observed a markedly weaker conditioning of camalexin accumulation in *sid2-1* than in Col-0, while *npr1-3* showed the weakest priming response (Figure 8, A and E).

Upon biological SAR induction, we previously also detected priming of the pathogen-induced biosynthesis of the NHP precursor Pip and of the immune signal SA (Návarová et al., 2012; Bernsdorff et al., 2016). We now observed that exogenous NHP alone was sufficient to significantly elevate the levels of Pip in the leaves (Figure 8, B and F). This direct effect of NHP on Pip accumulation was similarly detected in *sid2-1* but occurred to a reduced extent in *npr1-3*. Because of their defect in inducible Pip biosynthesis, the levels of Pip remained low in *ald1* mutant plants irrespective of the treatment applied (Figure 8, B and F). Notably, the NHP pretreatment also strongly primed the leaves of Col-0 plants to enhance the *Psm*-induced accumulation of Pip. As for camalexin, the priming of Pip accumulation by exogenous NHP was wild-type-like in *fmo1* plants, markedly reduced in *sid2-1*, and most strongly affected in *npr1-3* (Figure 8, B and F).

Quantification of the total levels of SA in our assays showed that NHP pretreatment also strongly primed Col-0 plants for an enhanced pathogen-triggered induction of SA biosynthesis (Figure 8, C and G). Although this priming effect was clearly detected on the level of free SA, it was more pronouncedly observed for the accumulation of the two SA conjugates SAG and SGE (Supplemental Figure S5). Exogenous NHP was able to induce wild-type-like priming of SA biosynthesis in the NHP biosynthetic mutants *ald1* and *fmo1*, but only weakly primed *npr1-3* for enhanced SA biosynthesis (Figure 8, C and G; Supplemental Figure S5). Interestingly, NHP pretreatment also significantly primed the leaves for an enhanced accumulation of Pip and SA in response to the mock-infiltration, indicating that NHP also primes responses to mechanical stress in Arabidopsis (Figure 8, B and C).

The metabolic response of Arabidopsis leaves toward *Psm* inoculation also involves elevation of the levels of the BCAAs Valine (Val), Leu, and Ile (Návarová et al., 2012; Zeier, 2013). We found that NHP pretreatment also sensitized plants for an enhanced *Psm*-induced accumulation of each of the three BCAAs (Figure 8, D and H; Supplemental Figure S6). Priming for enhanced BCAA accumulation was pronouncedly observed in *ald1* and *fmo1* plants, reduced in *sid2-1*, and absent in *npr1-3*, which parallels the tendency observed for the other metabolites. Moreover, exogenous NHP also directly induced a modest accumulation of the BCAAs in noninfested plants (Figure 8, D and H; Supplemental Figure S6).

### NHP fortifies SA-inducible immune responses in dependence of NPR1

**Figure 9:**
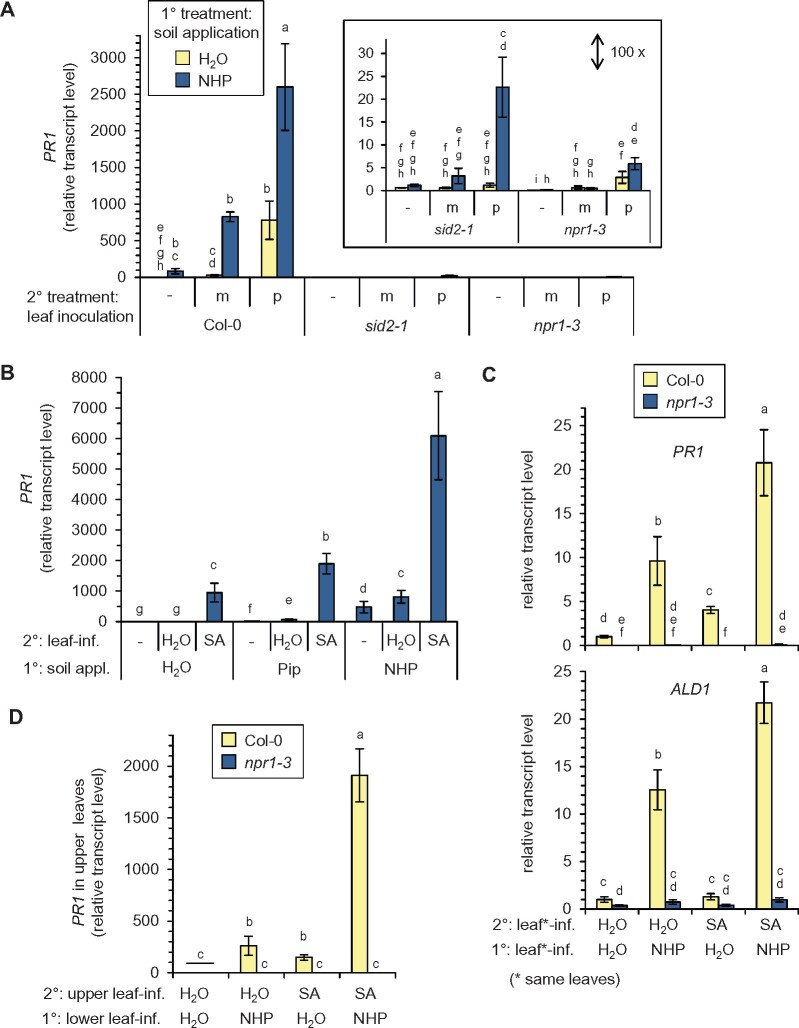
NHP and SA positively interact for the local and systemic induction of SAR-related gene expression. A, The NHP-induced priming of pathogen-triggered *PR1* expression is strongly dependent on SA biosynthesis and requires NPR1. Col-0, *sid2-1*, or *npr1-3* plants were supplied with 10 mL of 1 mM NHP or 10 mL of H_2_O via the soil (1° treatment), and leaves challenge-inoculated with *Psm* (p) or mock-infiltrated (m) with 10 mM MgCl_2_ 1 d later (2° treatment). The leaves of a third set of plants were left untreated (−). The transcript levels of *PR1* in the leaves were determined 12 h after the 2° treatment by RT-qPCR analysis (*n* = 4). Transcript values are given relative to the mean value of the Col-0 control samples (1° treatment H_2_O/no 2° treatment). B, NHP supplied via the soil primes the foliage for enhanced SA-induced *PR1* gene expression. Col-0 plants were 1°-treated with 1 mM NHP, 1 mM Pip, or H_2_O (10 mL each) via the soil. One day later, three leaves were infiltrated with a 0.5 mM SA solution or with H_2_O (2° treatment). The leaves of a third set of plants were left untreated (−). Leaf *PR1* transcript levels were determined 4 h after the 2° treatment (*n* = 4) and are given relative to the mean of the 1°-H_2_O- and 2°-H_2_O-treated samples. C, Leaves treated with exogenous NHP are primed for enhanced SA-inducible *PR1* and *ALD1* expression. Three leaves of Col-0 or *npr1-3* plants were infiltrated with 1 mM NHP (or H_2_O; 1° treatment) and the same leaves infiltrated one day later with 0.5 mM SA (or H_2_O; 2° treatment). Leaf *PR1* (top) and *ALD1* (bottom) transcript levels were determined 4 h after the 2° treatment (*n* = 4) and are given relative to the mean of the 1°-H_2_O- and 2°-H_2_O-treated samples. D, A local leaf application with NHP primes distant leaves for enhanced SA-induced *PR1* expression. Three lower rosette leaves of Col-0 or *npr1-3* plants were infiltrated with 1 mM NHP (or H_2_O; 1° treatment) and three upper leaves infiltrated one day later with 0.5 mM SA (or H_2_O; 2° treatment). Leaf *PR1* transcript levels were determined 4 h after the 2° treatment (*n* = 4) and are given relative to the mean of the 1°-H_2_O- and 2°-H_2_O-treated samples. Different letters denote significant differences (*P* < 0.05, Kruskal–Wallis *H* test).

Our previous results showed that exogenous Pip primes SA-inducible gene expression in dependence of functional FMO1 (Bernsdorff et al., 2016). We thus tested whether the pathogen-triggered expression of the SA-inducible gene *PR1* would be primed by exogenous NHP supplied via the soil. As shown in the above experiments (Figures 4A and 5), NHP pretreatment alone was sufficient to markedly elevate *PR1* transcript levels (Figure 9A). Moreover, exogenous NHP strongly primed the *Psm*-induced expression of *PR1* and also had a positive influence on the *PR1* transcript levels detected in mock-infiltrated leaves (Figure 9A; Supplemental Figure S7). The leaf transcript levels of *PR1* thereby paralleled the levels of SA totally accumulating in the corresponding leaves (Figures 8, C and G and 9A). Exogenous NHP was not able to induce *PR1* expression in *sid2-1* and *npr1-3*, indicating that the direct induction of *PR1* by NHP requires an intact SA signaling pathway (Figure 9A). In addition, the priming of *PR1* expression in response to the *Psm*-inoculation and the mock-infiltration was fully blocked in *npr1-3*, suggesting that *PR1* expression under all the applied conditions proceeds via NPR1 (Figure 9A). Furthermore, NHP-mediated priming of *PR1* was strongly dependent on inducible SA biosynthesis, since only a quantitatively modest (but significant) elevation of *PR1* levels was detected in NHP-pretreated and *Psm*-inoculated *sid2-1* plants (Figure 9A).

To further study the interaction of SA and NHP in defense gene expression, we exogenously supplied Col-0 plants with each of the immune-active metabolites individually or with a combination of both substances. In a first assay, plants were pretreated with H_2_O, 1 mM Pip, or 1 mM NHP via the soil, and their leaves one day later were either infiltrated with a 0.5 mM SA solution, infiltrated with H_2_O (mock-infiltration), or left untreated. Four hours after the second treatment, leaves were sampled for the assessment of *PR1* expression. As observed previously (Bernsdorff et al., 2016), leaf treatment with SA was sufficient to induce *PR1* expression, and pretreatment with Pip enhanced the SA-triggered expression of *PR1* (Figure 9B). Compared to the Pip application, pretreatment of plants with NHP caused a more intense induction of *PR1* transcript levels and further mediated a stronger priming of SA-induced *PR1* expression (Figure 9B). To discriminate potential local and systemic priming effects of NHP, we applied NHP to plants via leaf infiltration and 1 d later treated the same (Figure 9C; Supplemental Figure S8) or distant leaves (Figure 9D) with SA. Again, exogenous application of NHP was sufficient to induce *PR1* expression in both the treated and distant leaves (Figures 9, C and D). Moreover, local leaf-treatment with NHP primed both the treated and the distant leaves for an enhanced SA-triggered *PR1* expression. Thereby, we generally observed that the systemic priming effect was stronger than the local effect (Figure 9, C and D). Notably, the priming of SA-inducible *PR1* expression fully depended on a functional *NPR1* gene. This was observed for the soil and the leaf treatment modes of NHP and concerned both the local and the systemic NHP-mediated priming responses (Figure 9, C and D; Supplemental Figure S8).

In contrast to *PR1*, the expression of *ALD1* was primed in a partially SA-independent manner in plants exhibiting biologically induced SAR (Bernsdorff et al., 2016). To examine the interplay of NHP and SA in context with the expression of a gene with partially SA-independent induction characteristics, we assessed *ALD1* transcript levels in our priming assay. Application of SA to leaves alone had no impact on the *ALD1* transcript levels, but, as shown before (Figures 4A and 5), NHP application was sufficient to activate *ALD1* expression (Figure 9C). Moreover, plants that experienced a combined NHP and SA treatment showed stronger *ALD1* expression than plants treated with NHP only. Both the direct induction of *ALD1* expression by NHP and the positive effect of NHP on SA-inducible *ALD1* expression depended on functional NPR1 (Figure 9C). Together, these analyses show that NHP and SA positively interact to mediate defense gene expression. Besides the direct effects of NHP on gene transcription (Figures 3–5), NHP primes plants for an enhanced responsiveness to SA (Figure 9). This holds true for the expression of genes with strong SA-dependent (*PR1*) and partial SA-independent (*ALD1*) regulation, and both the direct and the priming effects mediated by NHP depended on the transcriptional coregulator NPR1.

## Discussion

Our previous results provided genetic and biochemical evidence that the endogenous, pathogen-triggered accumulation of NHP induces SAR in Arabidopsis (Hartmann et al., 2018). The aim of this study was to obtain information about the mode of action of NHP in SAR activation. To this end, we primarily used exogenous application of NHP by different treatment modes and experimental setups in Arabidopsis. On the basis of recent findings from our own and other laboratories, this study supports several previous hypotheses and provides valuable insights into the regulatory principles of NHP-induced SAR.

### NHP directly triggers the systemic transcriptional response of plants that is associated with SAR

**Figure 10:**
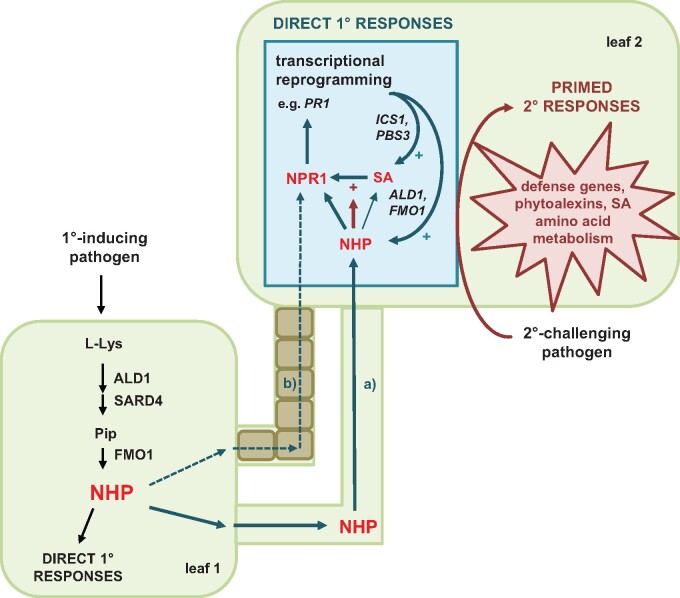
Simplified model for the establishment of SAR in response to localized pathogen inoculation. NHP that accumulates through pathogen attack in an inoculated leaf (leaf 1) can move (Route a) to distant leaves (leaf 2) and induce a direct transcriptional response that is fortified by accumulating SA and largely depends on NPR1. In parallel, NHP might also trigger SAR signaling from leaves 1 to 2 independent of its own movement (Route b). NHP accumulation during SAR also primes plants for enhanced response activation if a subsequent challenge infection should occur. NHP-induced defense priming is amplified by SA and depends to a predominant extent on NPR1 (for further details please refer to the text). Arrows in cyan indicate direct systemic responses; arrows in dark red indicate priming effects.

This study shows that elevated levels of NHP, as achieved by exogenous treatment of Arabidopsis with physiological doses of the authentic compound, are sufficient to directly induce a substantial transcriptional reprogramming of leaves that is associated with the upregulation of almost 1,900 genes (Figure 3). This NHP-triggered response is highly reminiscent of the transcriptional reprogramming that occurs in the distant leaves of locally pathogen-inoculated Arabidopsis plants during biological SAR induction (Figure 3C), which strictly depends on endogenous NHP biosynthesis via ALD1 and FMO1 (Gruner et al., 2013; Bernsdorff et al., 2016; Hartmann et al., 2018). Therefore, NHP is a necessary and sufficient signal to trigger transcriptional reprogramming in the course of SAR establishment (Figure 10).

In a quantitatively less pronounced manner, Arabidopsis also responds to the NHP biosynthetic precursor Pip (Figure 3B; Supplemental Figure S2). Notably, the Pip-induced transcriptional response strictly depends on functional FMO1, which catalyzes NHP biosynthesis from Pip via N-hydroxylation (Hartmann et al., 2018). Together with the high overlap of the NHP and Pip responses, this supports the notion that NHP is the actual mediator of the transcriptional SAR response and Pip essentially functions as a metabolic precursor that on its own is not immune-active. The less pronounced transcriptional response to exogenous Pip compared to exogenous NHP might be explained by the necessity of Pip-to-NHP conversion after Pip feeding while NHP feeding directly provides the SAR-active metabolite. In addition, it might also relate to different root uptake or shoot translocation characteristics of the two supplied metabolites within the plant.

The qualitative evaluation of the NHP- and SAR-induced transcriptional response indicates that NHP upregulates genes involved in distinct stages of plant immune signaling (Table 1; Bernsdorff et al., 2016). NHP- and SAR-upregulated genes include genes coding for immune receptor proteins such as RLKs, RLPs, and NLRs that perceive pathogen-derived and damage-associated molecules (Zipfel, 2014). NHP, therefore, enhances the surveillance system of plants during SAR, which will allow a more effective recognition of pathogens. To our knowledge, it has not yet been reported whether the knockout of an individual immune receptor would lead to complete SAR loss. However, transgenic Arabidopsis plants with elevated expression of distinct members of RLK families such cysteine-rich protein kinases (CRKs) or lectin-receptor-like kinases showed enhanced SAR responses or constitutively activated immunity (Acharya et al., 2007; Wang et al., 2015; Luo et al., 2017). A concerted NHP-regulated expression of many different immune receptors that recognize distinct bacterial, oomycete, or fungal pathogens might be one reason why SAR-induced plants exhibit broad-spectrum immunity against several pathogen types.

NHP also activates many genes involved in signal transduction downstream of pathogen perception, which function in protein phosphorylation, Ca^2+^-related signaling, and stress-inducible transcriptional regulation (Table 1). CDPKs act as Ca^2+^ sensors that decode and translate Ca^2+^ elevations into enhanced protein kinase activity and subsequent downstream signaling events (Harmon et al., 2000). The Arabidopsis CDPK genes *CPK5* and *CPK6* are, among others, induced by NHP (Supplemental Table S3). While *cpk5* and *cpk6* single mutants showed a wild-type-like SAR upon *P. syringae* inoculation, a *cpk5 cpk6* double mutant proved to be SAR-deficient. In addition, a *CPK5* overexpressing line induced *ALD1* and *FMO1* expression, exhibited constitutively elevated levels of NHP, and showed a *FMO1*-dependent increase in basal pathogen resistance (Guerra et al., 2020). Moreover, the MAPK gene *MPK3*, which contributes together with its putative paralog *MPK6* to the efficiency of biologically-induced SAR (Beckers et al., 2009; Wang et al., 2018), is induced by NHP. Conversely, a locally sustained activation of MAPK activity in transgenic Arabidopsis triggered Pip and NHP generation and induced SAR in distant tissue (Wang et al., 2018). MPK3 and MPK6 directly phosphorylate the transcription factor WRKY33 (Mao et al., 2011), which also exhibits increased expression after NHP treatment. In fact, the WRKY family represents the class of transcriptional regulators upregulated most widely in Arabidopsis by SAR-inducing treatments such as exogenous NHP application, treatment with the priming-activating chemical S-methyl-1,2,3-benzothiadiazole-7-carbothioate (BTH), and *P. syringae* inoculation (Table 1; Wang et al., 2006; Bernsdorff et al., 2016). WRKY33 directly binds to the *ALD1* promoter and positively influences NHP biosynthesis and SAR activation (Wang et al., 2018). These examples illustrate that NHP-induced signaling components such as CDPKs, MAPKs, and WRKYs can provide positive feedback on NHP biosynthesis, so that signal amplification loops for the activation of SAR are realized.

The biosynthesis and signaling pathways of the two key metabolic SAR regulators NHP and SA are closely intertwined (Hartmann and Zeier, 2019; Ding and Ding, 2020). Increasing levels of NHP trigger the expression of the genes required for inducible SA (*ICS1, EDS5*, and *PBS3*) and NHP biosynthesis (*ALD1*, *SARD4*, and *FMO1*; Figures 4A and 5). Moreover, a common set of proteins exists that both regulate NHP and SA biosynthesis as well as their downstream action. These factors include the two lipase-like proteins EDS1 and PAD4, the transcriptional regulators SARD1 and CALMODULIN-BINDING PROTEIN 60g, the transcriptional coregulator NPR1, and MILDEW RESISTANCE LOCUS O2 (Feys et al., 2001; Wang et al., 2011; Sun et al., 2015; Gruner et al., 2018; Hartmann et al., 2018; Sun et al., 2018). Notably, the transcription of each of the respective genes is inducible by NHP (Figure 4A), which provides the basis for a key feedback amplification mechanism involved in SAR establishment.

In addition, NHP also strongly upregulates genes encoding enzymes involved in metabolic conversion of NHP and SA, namely UGT76B1, SA-3-hydroxylase (S3H), and SA-5-hydroxylase (S5H; Figure 4A). It was shown recently by in vitro and in planta analyses that the glycosyltransferase UGT76B1 catalyzes the simultaneous glycosylation of SA and NHP to their respective β-glucosides SAG and NHPG (Bauer et al., 2021; Cai et al., 2021; Holmes et al., 2021; Mohnike et al., 2021). Although knock-out of *UGT76B1* in Arabidopsis resulted in enhanced basal NHP levels and an NHP-driven, constitutive SAR, overexpression of UGT76B1 abrogated pathogen-inducible NHP accumulation and SAR. This demonstrated an important function for UGT76B1 in regulating the homeostasis of active NHP and SA to balance the plant immune status (Bauer et al., 2021). SA homeostasis is further mediated by the 2-oxoglutarate-dependent dioxygenases S3H and S5H, which catalyze the hydroxylation of SA to 2,3- and 2,5-dihydroxybenzoic acid, respectively (Zhang et al., 2013; 2017). NHP accumulating after pathogen inoculation thereby not only regulates its own homeostasis but also promotes the metabolic inactivation of its signaling partner SA. This could avoid an over-activation of plant defenses and/or regulate the timing of SAR. Our finding that application of NHP to leaves not only elevates unconjugated SA but also the supposedly inactive glucose conjugates SAG and SGE in local and systemic leaf tissue supports this hypothesis (Figure 6; Supplemental Figures S3 and S4).

### NHP primes plants for a fortified induction of plant defense responses in the course of a challenge infection

Elevated levels of NHP directly trigger substantial transcriptional and metabolic reprogramming of leaves (Figures 4–6). Moreover, increased NHP levels due to pathogen inoculation or exogenous treatment equip plants with another, indirect defensive capacity: NHP primes plants for the boosted activation of defenses when facing a future pathogen challenge (Figure 10). We have previously characterized this priming effect in context with the pathogen-induced SAR response. Defense priming associated with biological SAR strictly depended on functional *ALD1* and *FMO1* genes, while a detectable but attenuated priming was observed in *sid2* plants (Návarová et al., 2012; Bernsdorff et al., 2016). This indicated that endogenously accumulating NHP orchestrates SAR-associated priming, while SA further fortifies this NHP-triggered conditioning. In addition, we have previously shown that exogenous Pip triggers priming in dependence of *FMO1*, suggesting that Pip to NHP conversion is necessary for the Pip-inducible priming effect (Bernsdorff et al., 2016; Hartmann et al., 2018). In this study, we directly show that NHP confers strong defense priming to Arabidopsis Col-0 plants. The nature of responses for which plants are primed after NHP application match the responses previously reported for biological and Pip-induced priming: accumulation of camalexin, biosynthesis of SA, generation of Pip, and activation of SAR-related gene expression. In addition, we have shown here that NHP also conditions plants for the pathogen-induced accumulation of the BCAAs Val, Leu, and Ile (Figures 8 and 9A; Supplemental Figures S5 and S6). The NHP-deficient *ald1* and *fmo1* mutants that lack biological priming regain priming-competency when exogenously supplied with NHP, supporting the previous assumption that their dysfunctionality in biological priming is due to their defects in endogenous NHP accumulation (Figure 8; Supplemental Figures S5 and S6). Moreover, albeit not fully absent, the NHP-induced priming of camalexin, Pip, and BCAA accumulation was diminished in *sid2-1*, which corroborates that the induction of SA biosynthesis fortifies the NHP-triggered priming response (Figure 8; Supplemental Figures S5 and S6).

The quantitative manifestation of a primed response in preconditioned plants may be described by a biphasic curve (Bruce et al., 2007). A first stress exposure (in our experimental setups associated with endogenous NHP production or equal with exogenous NHP feeding) induces the response to a certain level, and a second stress (in our setups the “pathogen challenge”) triggers a further, potentially amplified induction of the response. Eventually, the response induction will be higher in magnitude in a preconditioned plant than in an unprepared plant that only experiences the second but not the first stress exposure. In NHP-conditioned plants, such a course of events is observed for the accumulation of SA, Pip, and BCAAs. NHP-pretreatment already triggers these defenses to a small level, while the subsequent pathogen-challenge further boosts the responses to a much stronger level than in the unconditioned case (Figure 8; Supplemental Figures S5 and S6). For SA and Pip accumulation at least, this goes hand in hand with the direct induction of all the SA and Pip biosynthetic pathway genes by the NHP pretreatment (Figures 4A and 5).

Frequently, the priming phenomenon is defined more narrowly in the sense that primed responses are only those that are induced—in a more vigorous manner than usually—after the second stress exposure, but not yet directly activated by the first stress (Bruce et al., 2007). This scenario is fully represented by the camalexin response. NHP prepares plants for a strongly potentiated pathogen-elicited accumulation of camalexin, while it does not elevate the levels of the phytoalexin directly (Figure 8, A and E). How is the plant prepared for an enhanced accumulation of camalexin? On the one hand, NHP directly elevates the transcript levels of the camalexin biosynthetic genes *CYP71A12*, *CYP71A13*, and *PAD3*, while other pathway genes are not upregulated (Figure 4C; Mucha et al., 2019). Therefore, a partial activation of the biosynthetic pathway takes place at the level of transcription. This is not enough to directly induce accumulation of camalexin but is obviously sufficient to condition plants for a boosted stimulus-triggered response. On the other hand, NHP increases the transcript levels of genes involved in the kinase-mediated regulation of camalexin accumulation. For example, it was recently shown that CPK5/6 and MPK3/6 cooperatively regulate camalexin biosynthesis by differentially phosphorylating the WRKY33 transcription factor (Zhou et al., 2020). As the SA, Pip, and BCAA responses that follow on the combined NHP-preconditioning and pathogen-challenge treatments are much higher than the sum of the respective responses to the individual pre- and challenge-treatments, priming in the above-defined narrower sense is also a major factor in these metabolic responses (Figure 8; Supplemental Figures S5 and S6).

Previous findings also indicate that elevated NHP conditions Arabidopsis for a timely execution of the hypersensitive cell death response in interactions with avirulent *P. syringae* or with the compatible oomycete *Hpa* Noco2, which otherwise does not elicit cell death (Chen et al., 2018; Hartmann et al., 2018). The HR counteracts infection of plants by biotrophic or hemibiotrophic pathogens (Cui et al., 2015). Our RNA-seq study showed that NHP upregulates several cell death-related genes, such as *ACCELERATED CELL DEATH6* or metacaspases (Rate et al., 1999; He et al., 2008; Watanabe and Lam, 2011), which might be involved in the NHP-mediated promotion of the HR (Figure 4B).

The NHP-induced defense priming during SAR is also evident at the level of defense-related gene expression (Figure 9A; Návarová et al., 2012; Bernsdorff et al., 2016). Increasing evidence indicates that plant defense gene expression is under epigenetic control and involves DNA methylation and histone modifications, which are closely linked to accessible (transcriptionally active) and inaccessible chromatin states (Ramirez-Prado et al., 2018). A recent preprint reports a correlation between the speed of transcriptional upregulation in different scenarios of Arabidopsis receptor-mediated immunity and accessible chromatin regions in promoters of NHP (*ALD1*, *SARD4*, and *FMO1*) and SA (*ICS1* and *PBS3*) biosynthetic genes (Ding et al., 2020). Moreover, it was shown that the histone demethylase JMJ14 positively affects the induction of NHP biosynthetic genes, accumulation of Pip, and SAR establishment (Li et al., 2020). An interesting but still unresolved question is whether epigenetic control mechanisms are involved in the NHP-mediated priming response during SAR.

### NHP positively interacts with SA to mediate SAR

The NHP precursor Pip primed Arabidopsis wild-type but not *fmo1* mutant plants for a fortified SA-triggered expression of *PR1* (Bernsdorff et al., 2016). On this basis, we previously hypothesized that NHP would sensitize plants to more strongly respond to SA (Hartmann and Zeier, 2019). This hypothesis was now confirmed by a more direct experimental approach that used NHP instead of Pip supply to plants (Figure 9, B–D). Interestingly, when NHP was locally applied to specific leaves, we observed a particularly strong conditioning of SA-inducible *PR1* expression in distal leaves (Figure 9D), emphasizing the character of NHP as a systemic resistance activator. While *PR1* is a paradigm example for an SA inducible gene, *ALD1* is not induced upon SA treatment alone (Figure 9C; Bernsdorff et al., 2016). However, elevated NHP conferred the ability to increase *ALD1* transcript levels in response to SA (Figure 9C). The positive crosstalk of NHP and SA in systemic immunity therefore expresses itself on several levels: First, NHP induces SA biosynthesis and sensitizes plants for enhanced pathogen-triggered SA production (Figures 4A, 6, and 8). Second, NHP primes for enhanced SA-inducible expression of defense genes (Figure 9, B–D). And third, SA strongly fortifies the NHP-triggered transcriptional SAR response (Figure 3A; Bernsdorff et al., 2016). The positive interplay between NHP and SA in plant immunity is also reflected in an increased susceptibility to infection by *P. syringae* of both NHP- and SA-deficient *ald1 sid2* or *fmo1 sid2* double mutants compared to the respective single mutants that only lack one of the two immune regulators (Bernsdorff et al., 2016; Liu et al., 2020).

### NPR1 is a main downstream mediator of NHP-inducible defenses (which may have both SA-dependent and SA-independent features)

The ability of NHP to induce immunity in plants is largely dependent on a functional SA biosynthetic pathway (Hartmann et al., 2018; Liu et al., 2020; Bauer et al., 2021). Nevertheless, with respect to NHP-triggered SAR activation, transcriptional reprogramming and induction of defense priming, we generally observed modest but significant responses in the SA-induction-deficient *sid2* plants (Figures 1, 3A, 5B, and 7; Hartmann et al., 2018). The *ICS1*-defective *sid2* mutant is unable to significantly elevate SA levels in response to *Psm* inoculation in the local or systemic leaf tissue and contains reduced basal levels of SA (Figure 7; Bernsdorff et al., 2016). It is unlikely in our opinion that these reduced basal SA levels markedly contribute to an inducible immune response such as SAR. Under this premise, NHP evidently triggers a dominant SA-dependent and a minor SA-independent defense pathway to SAR. The transcriptional co-activator NPR1 is a key downstream mediator of SA signaling, functions as a bonafide receptor of SA and is required for pathogen-induced SAR in Arabidopsis (Wu et al., 2012; Ding et al., 2018; Spoel, 2019; Ding et al., 2020; Ding and Ding, 2020). We have previously reported that SAR induced by the application of exogenous Pip in Arabidopsis was diminished to a greater extent in *npr1* than in *sid2* mutant plants (Návarová et al., 2012). This already suggested that NPR1 might be an important downstream mediator of NHP-inducible immunity. Here, we show that acquired resistance induced by NHP-pretreatment, either via the soil or the leaves, to *P. syringae* or *Hpa* infection, is virtually absent (*P. syringae*; Figure 1) or severely compromised (*Hpa*; Figure 2) in *npr1* mutants. In particular, the residual SAR response observed in the upper leaves of *sid2* plants pretreated in lower leaves with NHP was absent in both *npr1* and a *sid2 npr1* double mutant (Figure 1C). Moreover, the NHP-induced transcriptional reprogramming and the priming response were both more strictly compromised in *npr1* than in *sid2* mutant plants (Figures 3A and 8). For example, the expression of the *ALD1* gene, which is partially induced in *sid2* during *P. syringae*-induced SAR (Bernsdorff et al., 2016), was elevated in *sid2* by exogenous NHP treatment, but not in *npr1* and *sid2 npr1* (Figure 5B). These findings indicate that NHP-triggered immunity, including the residual SA-independent NHP response that occurs in *sid2*, is to a major part NPR1-dependent (Figure 10).

Our results on the requirement of NPR1 for NHP-inducible immunity are consistent with other recent findings. Simultaneous mutational defects in the Arabidopsis calmodulin-binding transcription factors CAMTA1, CAMTA2, and CAMTA3, which act as negative regulators of NHP and SA biosynthetic genes, result in elevated basal levels of Pip and NHP, as well as in *FMO1*-, *ALD1*-, and *NPR1*-dependent autoimmunity and dwarfism (Kim et al., 2020; Sun et al., 2020). Although NHP levels were not measured in their study, the results of Kim et al. (2020) show that increased amounts of Pip in the *camta1/2/3* triple mutant background are associated with increased total amounts of NPR1 protein in leaf extracts. Consistently, *NPR1* transcript levels are increased in response to NHP or Pip treatments (Figure 4A; Hartmann et al., 2018). Thus, a possible scenario that requires future validation is that NHP increases the active levels of the SA receptor NPR1 and thereby ensures enhanced SA-mediated plant immunity. Moreover, in parallel to our study, Liu et al. (2020) found that *NPR1* is required for the activation of acquired resistance to *Hpa* by exogenously applied NHP. These authors also examined whether NHP would directly bind to NPR1 and thereby mediate resistance, but a competitive binding assay based on size exclusion chromatography argued against this hypothesis (Liu et al., 2020). Therefore, further research is required to elucidate the molecular mechanisms of how NHP mediates SAR in cooperation with SA and NPR1.

Notably, the actions of NHP as an endogenous mediator of SAR, SAR-associated transcriptional reprogramming, and defense priming are highly reminiscent of the effects of the synthetic benzothiadiazole derivative BTH on plants. For example, BTH was found to induce a strong, SAR-like transcriptional response which largely depends on functional *NPR1* (Wang et al., 2006; Gruner et al., 2013), triggers NPR1-dependent defense priming (Kohler et al., 2002), and induces NPR1-mediated SAR to pathogens (Uknes et al., 1992). Considering these similarities between NHP and BTH, the often-used expression “SA analogue” for BTH appears rather vague and imprecise.

### NHP is a mobile, systemic immune regulator that can travel independently of active SA signaling

Chen et al. (2018) had observed previously that exogenous application of NHP to lower leaves of Arabidopsis *fmo1* plants led to an increase of the levels of glycosylated NHP in upper leaves. Moreover, transient *Agrobacterium tumefaciens*-mediated expression of Arabidopsis *ALD1* and *FMO1* in proximal leaflets of tomato leaves resulted in increased NHP levels of distal leaflets and a concomitant heightened immunity against *P. syringae* infection (Holmes et al., 2019). It was inferred that NHP or an NHP conjugate could travel from treated to distant leaves during SAR. Further, in a detailed time course analysis, NHP started to accumulate in the distant leaves of locally *Psm*-inoculated Arabidopsis plants before rises of Pip and SA were detectable (Hartmann et al., 2018). This suggested that NHP generated in inoculated leaves could travel as a primary mobile signal to distant leaves at the onset of biologically induced SAR. Moreover, NHP strongly accumulated in the phloem sap collected from both pathogen-inoculated and distant leaves of cucumber plants, which indicated a function of NHP as a phloem-mobile immune signal (Schnake et al., 2020).

The results of this study substantiate that NHP acts as a mobile inducer of SAR. We used exogenous application of D_9_-NHP or NHP and leaf inoculation by *Psm* in different genetic backgrounds to examine leaf-to-leaf long-distance movement of NHP in Arabidopsis (Figures 6 and 7). After (D_9_)-NHP application to lower leaves, a significant portion of the (D_9_)-NHP was detected in upper, untreated leaves, indicating a substantial ability of leaf-to-leaf movement for (D_9_)-NHP. This leaf-to-leaf translocation also occurred in *sid2* and in *npr1* and was therefore independent of intact SA signaling (Figure 6; Supplemental Figure S4). In the interaction of *Psm* with Arabidopsis, a marked accumulation of NHP was observed for Col-0, *sid2*, and *npr1* plants in the distant leaves (Figure 7). The high leaf-to-leaf mobility of NHP suggests that a significant part of the systemically accumulating NHP is due to import from the inoculation sites also in the biological interaction. For *sid2* and *npr1*, this might be the dominant route of how NHP levels are enhanced in the distant leaves in response *to P. syringae*, because the systemic transcriptional response in these leaves that is required for a de novo NHP synthesis is comparatively weak, and at the same time, the local production of NHP is enhanced (Figures 3A and 7; Gruner et al., 2013; Bernsdorff et al., 2016; Hartmann et al., 2018). For the wild-type, a marked additional contribution of a de novo synthetic route is likely, because the NHP biosynthetic genes are strongly upregulated in the distant SAR tissue as a part of the abovediscussed NHP-driven amplification loop (Figures 4A and 5A; Bernsdorff et al., 2016).

We also detected a partial metabolic conversion of (D_9_)-NHP to the β-glucoside (D_9_)-NHPG and the glucose ester (D_9_)-NHPGE in the treated leaves. The conversion of NHP to NHPG, which is mediated by the UDP-glucose-dependent glycosyltransferase UGT76B1 (Bauer et al., 2021), was attenuated in both *sid2* and *npr1* mutant plants (Figure 6; Supplemental Figure S4). This is consistent with the promotion of pathogen-induced NHPG accumulation by SA signaling, and with a consequent over-accumulation of unconjugated NHP and of NHPGE in *sid2* and *npr1* after *P. syringae* attack (Hartmann et al., 2018; Bauer et al., 2021). In this context, it is noteworthy that our present metabolite analyses analogously indicate a NPR1-stimulated glycosylation of SA to SAG. While SA and SGE locally over-accumulate in *npr1* mutants in response to *P. syringae*, SAG formation tended to be reduced (Figure 7). UGT76B1 represents the common β-glycosylating enzyme for both NHP and SA (Bauer et al., 2021), and a recently reported dependency of *UGT76B1* expression on *NPR1* might be causative for these metabolic imbalances detected in *npr1* plants (Liu et al., 2020).

As locally applied (D_9_)-NHP also enhanced the (D_9_)-NHPG and (D_9_)-NHPGE levels systemically (Figure 6), a leaf-to-leaf transport of NHP glucose conjugates is possible as well. However, whether the NHP conjugates are (enzymatically) reconvertible into NHP is far from clear. The SAR-defective phenotype of a UGT76B1-overexpressor line, which is deficient in NHP accumulation because of NHPG over-production, supports the concept that free NHP is the immune active form and NHPG is an inactive conjugate (Bauer et al., 2021; Cai et al., 2021).

Finally, the (D_9_)-NHP-treatment also induced accumulation of SA and its glucose conjugates SAG and SGE in both treated lower and untreated upper leaves. Notably, the systemic induction of SA biosynthesis by exogenous (D_9_)-NHP did not require NPR1 (Figure 6; Supplemental Figures S3 and S4). In response to *P. syringae* attack, accumulation of SA, SAG, and SGE in the distant leaves of *npr1* plants was clearly discernable, albeit much lower than in the wild-type (Figure 7). Consistently, *P. syringae*-induced systemic increases of total SA levels in *npr1* mutants were observed in a previous study (Delaney et al., 1995). Although the major immune action of NHP unfolds in conjunction with NPR1, this suggests that an NHP-triggered and NPR1-independent signaling mode to some extent contributes to the systemic activation of SA biosynthesis in SAR (Figure 10).

## Conclusions

Together, we propose the following generalized and simplified scenario as a working model for the systemic induction of defense responses by NHP during biologically induced SAR (Figure 10): as part of a massive metabolic response triggered by microbial attack in inoculated leaves, l-Lys is converted by the pathogen-inducible ALD1, SARD4, and FMO1 enzymes into NHP. NHP strongly accumulates in the local leaves and moves, possibly via the phloem, to distant leaves (Route a). The concomitant rise of NHP in the systemic leaf tissue induces a transcriptional response that includes activation of NHP biosynthetic enzymes that further promotes NHP accumulation and action via de novo synthesis. In addition, SA biosynthetic genes are activated that result in the systemic accumulation of SA, which decisively fuels the SAR-associated transcriptional reprogramming. The strong accumulation of NHP in 1°-inoculated leaf tissue might also initiate long-distance signaling modes alongside NHP translocation, such as cell-to-cell signaling processes, which additionally contribute to the systemic activation of defenses (Route b). The major portion of systemic SAR signaling, apart from a modest activation pathway of SA biosynthesis, proceeds via the transcriptional co-activator NPR1. The transcriptional SAR response does not directly activate the full defense capacity of the plant but preconditions a primed state that prepares for a boosted activation of defenses in the case of a future challenge infection. The establishment of defense priming is triggered by elevated NHP and fortified by SA. During a pathogen challenge, NHP boosts SA-inducible and other responses in broad dependence of NPR1 to allow an effective, disease-preventing immune response.

## Materials and methods

### Plant materials and growth conditions

Individual Arabidopsis (*A. thaliana*) plants were cultivated in pots filled with a mixture of soil (Substrat BP3; Klasmann-Deilmann), vermiculite, and sand (8:1:1) in a controlled plant growth room. A 10-h-d (9 AM to 7 PM)/14-h-night cycle with a photon flux density of 100 μmol m^−2^ s^−1^ during the day and a relative humidity of 60% was applied. Day and night temperatures were set to 21°C and 18°C, respectively. Experiments were performed with 5-week-old plants.

The following Arabidopsis lines were used in the study: Col-0 (Nottingham Arabidopsis Stock Centre [NASC] ID: N1092), *ald1* (SALK_007673; Návarová et al., 2012), *fmo1* (SALK_026163; Mishina and Zeier, 2006), *sid2-1* (Nawrath and Métraux, 1999; Bernsdorff et al., 2016), *sid2-2* (N16438), *npr1-1* (N3726), *npr1-3* (N3802), and *pbs3-1* (SALK_018225). All mutants are in the Col-0 background. The *sid2-2 npr1-1* double mutant was generated by crossing *sid2-2* (Nawrath and Métraux, 1999), obtained from F. M. Ausubel (Harvard University, Boston, USA), and *npr1-1* (Cao et al. 1997, obtained from NASC) mutant plants. Homozygous F2 plants were identified by PCR (905 bp wild-type PCR product, no product for the *sid2-2* allele) using the primers *sid2-2*-F and *sid2-2*-R (Supplemental Table S5), and via a cleaved-amplified polymorphic sequence marker for *npr1-1* (primers *npr1-1*-NlaIII-F and *npr1-1*-NlaIII-R; Supplemental Table S5; Nishimura et al., 2003). To facilitate efficient seed production, inflorescences and leaves of 5–6-week-old homozygous mutant plants were sprayed with 1 mM SA. This procedure was repeated in the F3 generation after analyzing plants again to verify the double mutant genotype.

### Cultivation of plant pathogens and plant inoculation

*Psm* strain ES4326 and *Psm* expressing the luxCDABE operon from *Photorhabdus luminescens* (*Psm lux*) were cultivated at 28°C in King’s B medium as described (Bernsdorff et al., 2016; Gruner et al., 2018). Bacterial suspensions resulting from overnight cultured were washed three times with 10-mM MgCl_2_ and diluted to optical densities at 600 nm (OD_600_) of 0.005 (*Psm*) and 0.001 (*Psm lux*). *Psm* was used for inoculation experiments in context with the determination of metabolite contents and the analysis of gene expression (Figures 7–9A). The *Psm lux* strain was used for bacterial growth assays (Figure 1). Between 10 AM and 12 PM, bacterial solutions were uniformly infiltrated from the abaxial side into the leaves using needleless syringes. Control treatments involved mock-infiltrations of leaves with a 10 mM MgCl_2_ solution.

As detailed previously, *Hpa* isolate Noco2 was propagated on Col-0 plants (Hartmann et al., 2018). Plants were inoculated by spraying a suspension of 5 × 10^4^ sporangia per milliliter of H_2_O onto plants until the leaves were saturated. The inoculated plants were maintained on sealed trays with a transparent lid in a plant growth chamber (SE-41; Percival Scientific/CLF Plant Climatics, Wertingen, Germany) under the above-mentioned light and temperature conditions.

### Treatment with NHP (or D_9_-NHP) and resistance assays

NHP and D_9_-NHP were chemically synthesized according to a protocol of Murahashi and Shiota from piperidine and piperidine-D_11_ (448141; Sigma-Aldrich, St Louis, MO, USA), respectively (Murahashi and Shiota, 1987; Hartmann et al., 2018). NHP was either supplied to Arabidopsis via the soil or infiltrated into the leaves. For soil treatments (Figures 1A; 2; 3; 8; and 9, A and B), 10 mL of a 1 mM aqueous NHP solution was pipetted onto the soil of individually cultivated plants. Supply with 10 mL of H_2_O served as a control treatment (Hartmann et al., 2018). In two experiments (Figures 3B and 9B), a 1 mM solution of Pip was analogously applied. For leaf treatments (Figures 1, B and C; 5; 6; and 9, C and D) three rosette leaves per plant were infiltrated with a 1 mM NHP solution or with H_2_O. In the same manner (Figure 6), D_9_-NHP was applied (Hartmann et al., 2018).

For bacterial growth assays (Figure 1), three full-grown leaves of a plant were infiltrated 24 h after the pretreatment with bioluminescent *Psm lux*, leaf discs punched out of inoculated leaves, and bacterial growth assessed 60 h later as relative light units (rlus)/cm^2^ using a Sirius luminometer (Berthold Detection Systems GmbH, Pforzheim, Germany; Gruner et al., 2018). To estimate local resistance responses of NHP, the three NHP-preinfiltrated leaves were subsequently inoculated with bacteria (Figure 1B). To assess systemic responses, three lower rosette leaves were pretreated, and three upper (distant and systemic) leaves were inoculated (Figure 1C). Bacterial growth values were based on nine or more biological replicates, and each replicate value was determined from three inoculated leaves of a given plant.

The degree of *Hpa* infection was assessed as detailed previously (Hartmann et al., 2018). In brief, 1 d after the pretreatment, the leaf rosettes of plants were spray-inoculated with a suspension of sporangia (5 × 10^4^ mL^−1^) of *Hpa* isolate Noco2 as described. Seven days later, harvested leaves were stained with Trypan blue, destained with chloral hydrate solution, and leaf images captured with a photographic camera (Canon EOS 6D DSLR). Digital images were analyzed using the ImageJ software to determine the length of IH/cm^2^ leaf area and the number of oospores/cm^2^. The values of the depicted resistance parameters originate from evaluation of at least 10 leaf replicates from 6 different plants (Figure 2).

### Determination of metabolite levels

At the indicated times after (D_9_)-NHP-, *P. syringae*- or control-treatments (Figures 6–8), three leaves from two different plants were harvested, combined for one biological replicate, and immediately shock-frozen in liquid nitrogen. Metabolite levels in leaves were determined by gas chromatography–mass spectrometry (GC–MS)-based analyses according to a previous protocol with slight modifications (Hartmann et al. 2018; Bauer et al., 2021). Approximately 50 mg of the pulverized, frozen leaf sample was extracted twice with 1 mL of MeOH/50 mM sodium phosphate (pH 6.0; 80:20, v/v), and metabolite levels determined after derivatization by trimethylsilylation as previously detailed using an Agilent 7890A/5975C GC–MS system equipped with a Phenomenex ZB-35 (30 m × 0.25 mm × 0.25 µm) capillary column and MSD ChemStation software version E.02.01.1177 (Agilent Technologies, Santa Clara, CA, USA). The following selected ion chromatograms were used for the quantitative assessment of metabolites: NHP, NHPG, and NHPGE: *m*/*z* 172; D_9_-NHP, D_9_-NHPG, and D_9_-NHPGE (D_9_-NHPGE): *m*/*z* 181; Pip: *m*/*z* 156; SA, SAG: *m*/*z* 267; SGE: *m*/*z* 193; camalexin: *m*/*z* 272; Val: *m*/*z* 144; Leu and Ile: *m*/*z* 158. For absolute quantification, substance areas of the analytes were related to the following internal standards: NHP: D_9_-NHP (*m*/*z* 181) or 2-hydroxy-cyclohexanecarboxylic acid (2-CHC; *m*/*z* 273); D_9_-NHP: 2-CHC (*m*/*z* 273); Pip: D_9_-Pip (*m*/*z* 265); SA: D_4_-SA (*m*/*z* 271); (D_9_)-NHPG, (D_9_)-NHPGE, SAG, and SGE: salicin (*m*/*z* 268); camalexin: indole-3-propionic acid (*m*/*z* 202); Val, Leu, and Ile: norvaline (*m*/*z* 144). Generally, the metabolite levels for a given treatment and genotype represent the mean of four biological replicate samples.

### Analysis of gene expression by RT-qPCR analysis

To assess the expression of specific genes (Figures 5 and 9), samples were collected as described above for metabolite analysis. From approximately 50 mg of frozen leaf tissue, RNA isolation, cDNA synthesis, and RT-qPCR analysis were performed as detailed in Návarová et al. (2012). The *POLYPYRIMIDINE TRACT-BINDING PROTEIN 1* gene (At3g01150), which is nonresponsive to biotic stress, was used as a reference gene (Czechowski et al., 2005). The gene-specific primers used in this study are listed in Supplemental Table S5. As indicated in the figure legends, expression levels were given relative to the wild-type control values. Generally, the mean of four biological replicate samples is given.

### Assessment of defense priming

The assays to test for priming of immune responses by exogenous NHP (Figures 8 and 9) consisted of an inducing treatment with NHP or an H_2_O-control treatment as described above, and a subsequent secondary treatment.

To assess priming of pathogen responses (Figures 8 and 9A), individual plants were soil-treated with NHP or H_2_O, and 24 h later, three full-grown leaves per plant were challenge-inoculated with *Psm* or mock-infiltrated. The leaves of a third set of plants were not treated at all. The leaves were harvested 12 h later for metabolite determination and gene expression analyses as described above.

To examine the signal interactions between NHP and SA (Figure 9, B–D), plants were pretreated with NHP via soil- or leaf-application, and three leaves infiltrated 24 h later with an aqueous solution of SA (0.5 mM; pH 7.0) or with H_2_O was a control.

### Genome-wide analyses of the NHP transcriptional response by RNA-seq

Individual plants were watered with 10 mL of 1 mM NHP, 10 mL of 1 mM Pip, or 10 mL of H_2_O as described, and three leaves per plant harvested one day later for RNA-seq analyses. The detailed experimental setup is illustrated in Supplemental Table S1. The analysis is based on three independently conducted experiments.

Total RNA was extracted from the leaf sample replicates with the Plant RNeasy extraction kit (Qiagen, Hilden, Germany), and treated on-column (Qiagen, Germany) and in solution with RNA-free DNAse (New England Biolabs, Ipswich, MA, USA). Total RNA used for transcriptome analyses was quantified (Qubit RNA HS Assay, Thermo Fisher Scientific, Waltham, MA, USA) and the quality measured by capillary electrophoresis using the Fragment Analyzer and the “Total RNA Standard Sensitivity Assay” (Agilent Technologies, Inc. Santa Clara, CA, USA). All samples in this study showed good RNA Quality Numbers (RQN; mean = 7.8). The library preparation was performed according to the manufacturer’s protocol using the “VAHTS™ Stranded mRNA-Seq Library Prep Kit” for Illumina^®^. Briefly, 300-ng total RNA was used for mRNA capturing, fragmentation, the synthesis of cDNA, adapter ligation, and library amplification. Bead purified libraries were normalized and finally sequenced on the HiSeq 3000/4000 system (Illumina Inc. San Diego, CA, USA) with a read setup of 1 × 150 bp. The bcl2fastq tool was used to convert the BCL files to FASTQ files as well for adapter trimming and demultiplexing.

Data analyses on FASTQ files were conducted with CLC Genomics Workbench version 12.0.3 (QIAGEN, Venlo, NL, USA). The reads of all probes were adapter trimmed (Illumina TruSeq) and quality trimmed (using the default parameters: bases below Q13 were trimmed from the end of the reads, ambiguous nucleotides maximal 2). Mapping was done against the *A. thaliana* (TAIR10; May 25, 2017) genome sequence. The GO annotation gene_association.tair_2019-07-11 was used. After grouping of samples (three biological replicates each) according to their respective experimental condition, multigroup comparisons were made and statistically determined using the Empirical Analysis of DGE (version 1.1, cutoff = 5). The resulting *P*-values were corrected for multiple testing by FDR and Bonferroni correction. A *P*-value of ≤0.05 was considered significant.

The complete RNA-seq data of the NHP transcriptional response is provided as Supplemental Table S6 and the raw data deposited in the ArrayExpress database under the accession number E-MTAB-10230. Genes significantly upregulated (downregulated) in the Col-0 wild-type in response to NHP and Pip (*P* ≤ 0.05) and a fold-change ≥1.5 (≤0.67) were defined as NHP^+^ (NHP^−^) and Pip^+^ (Pip^−^) genes, respectively (Figure 3; Tables 1 and 2). Gene enrichment analysis was performed with the TAIR GO Term Enrichment Tool (https://www.arabidopsis.org/tools/go_term_enrichment.jsp), and the analyses of gene families were based on TAIR10 family annotation and published lists of gene families (Bernsdorff et al., 2016; Hartmann et al., 2018). Fisher’s exact test was used to test for significances of enrichment or depletion of gene categories or families in the defined gene groups. The additionally depicted RNA-seq data of the biological SAR response in wild-type plants (Figure 3C; Tables 1 and 2) is derived from experiments in which three lower rosette leaves of 5-week-old Col-0 plants were inoculated with *Psm* (OD_600_ = 0.005) or mock-treated, and three upper leaves harvested 2 d later for RNA-seq analysis (“SAR experimental dataset II” in Bernsdorff et al., 2016; ArrayExpress number E-MTAB-4151). The two datasets for the NHP- and biological SAR-responses were merged with the Microsoft Excel^®^ macro FIRe (Garcion et al., 2006).

### Statistical analyses

The number of biological replicates used for each experiment is indicated in the figure legends and in the individual paragraphs of the “Materials and methods” section. Statistical analyses were performed with the SPSS^®^ statistical software version 26 (IBM^®^ Corporation, Armonk, NY USA). To test for statistical differences between data subsets of bacterial growth and *Hpa* infection experiments (Figures 1 and 2), log_10_-transformed measuring values were subject to analysis of variance (ANOVA) and a post hoc Tukey’s HSD test (significance level *P* < 0.05; Hartmann et al., 2018). To test for differences in data subsets of metabolite and RT-qPCR-based gene expression experiments (Figures 7–9), nonparametric one-way ANOVA according to Kruskal–Wallis with stepwise step-down comparisons were applied on nontransformed data (significance level *P* < 0.05). Pairwise comparisons between two datasets (control versus treatment or genotype 1 versus genotype 2) were performed by a two-tailed Student’s *t* test in Microsoft Excel^®^ (Figures 5 and 6). The tendencies observed in the depicted experiments were validated in two or more independent experiments.

### Accession numbers

Sequence data from genes described in this article can be found in the Arabidopsis Genome Initiative or GenBank/EMPL databases under the following accession numbers: *ALD1* (At2g13810), *FMO1* (At1g19250), *SID2/ICS1* (At1g74710), *PBS3* (At5g13320), and *NPR1* (At1g64280). Other genes are listed in Supplemental Table S3.

## Supplemental data

The following materials are available in the online version of this article.

**Supplemental Figure S1.** Assessment of initial bacterial numbers in leaves after inoculation with *Psm lux* in the experimental settings presented in Figure 1.

**Supplemental Figure S2.** The transcriptional response to NHP is qualitatively similar to but quantitatively higher than the transcriptional response to Pip in Col-0 plants.

**Supplemental Figure S3.** Leaf-applied D_9_-NHP induces systemic SA accumulation independently from NPR1.

**Supplemental Figure S4.** Leaf-applied NHP translocates from treated to distant leaves, is partially glycosylated, and induces systemic SA accumulation in an *NPR1*-independent manner.

**Supplemental Figure S5.** Exogenous NHP primes plants for enhanced stimulus-induced SA biosynthesis.

**Supplemental Figure S6.** Exogenous NHP primes plants for enhanced pathogen-induced BCAA accumulation.

**Supplemental Figure S7.** NHP applied via the soil primes the Arabidopsis foliage for enhanced *PR1* expression in an NPR1-dependent manner.

**Supplemental Figure S8.** Arabidopsis leaves treated with exogenous NHP are primed for enhanced SA-inducible *PR1* expression.

**Supplemental Table S1.** Experimental setup to examine the transcriptional response to NHP by RNA-seq.

**Supplemental Table S2.** Occurrence of NHP^+^ and SAR^+^ genes in further groups of GO terms and gene families.

**Supplemental Table S3.** Selected immune-related genes upregulated by NHP.

**Supplemental Table S4.** Occurrence of NHP^−^ and SAR^−^ genes in further groups of GO terms.

**Supplemental Table S5.** Primers used in this study.

**Supplemental Table S6.** Full RNA-seq dataset of the transcriptional response to NHP in Col-0, *sid2-1*, and *npr1-3.*

## Supplementary Material

kiab166_Supplementary_Data

## Notes

I.Y., M.M., M.H., T.Z., and J.K. performed the experiments and contributed to data evaluation. P.P., K.K., and J.Z. performed RNA-sequencing analyses and evaluated the expression data. C.T. and C.G. generated the sid2-2 npr1-1 double mutant. J.Z. designed the research, contributed to data evaluation, and wrote the manuscript.

The author responsible for distribution of materials integral to the findings presented in this article in accordance with the policy described in the Instructions for Authors (https://academic.oup.com/plphys/pages/general-instructions) is: Jürgen Zeier (juergen.zeier@hhu.de).
