# Supplementary material for: The mobile SAR signal N-hydroxypipecolic acid induces NPR1-dependent transcriptional reprogramming and immune priming
Source: Plant Physiol. 2021 Apr 19;186(3):1679–705. doi: 10.1093/plphys/kiab166 (PMC8260123; doi:10.1093/plphys/kiab166)
Supplement: kiab166_Supplementary_Data [file kiab166_supplementary_data.zip › kiab166-suppl_data/pp.00039.2021-s01.pdf]

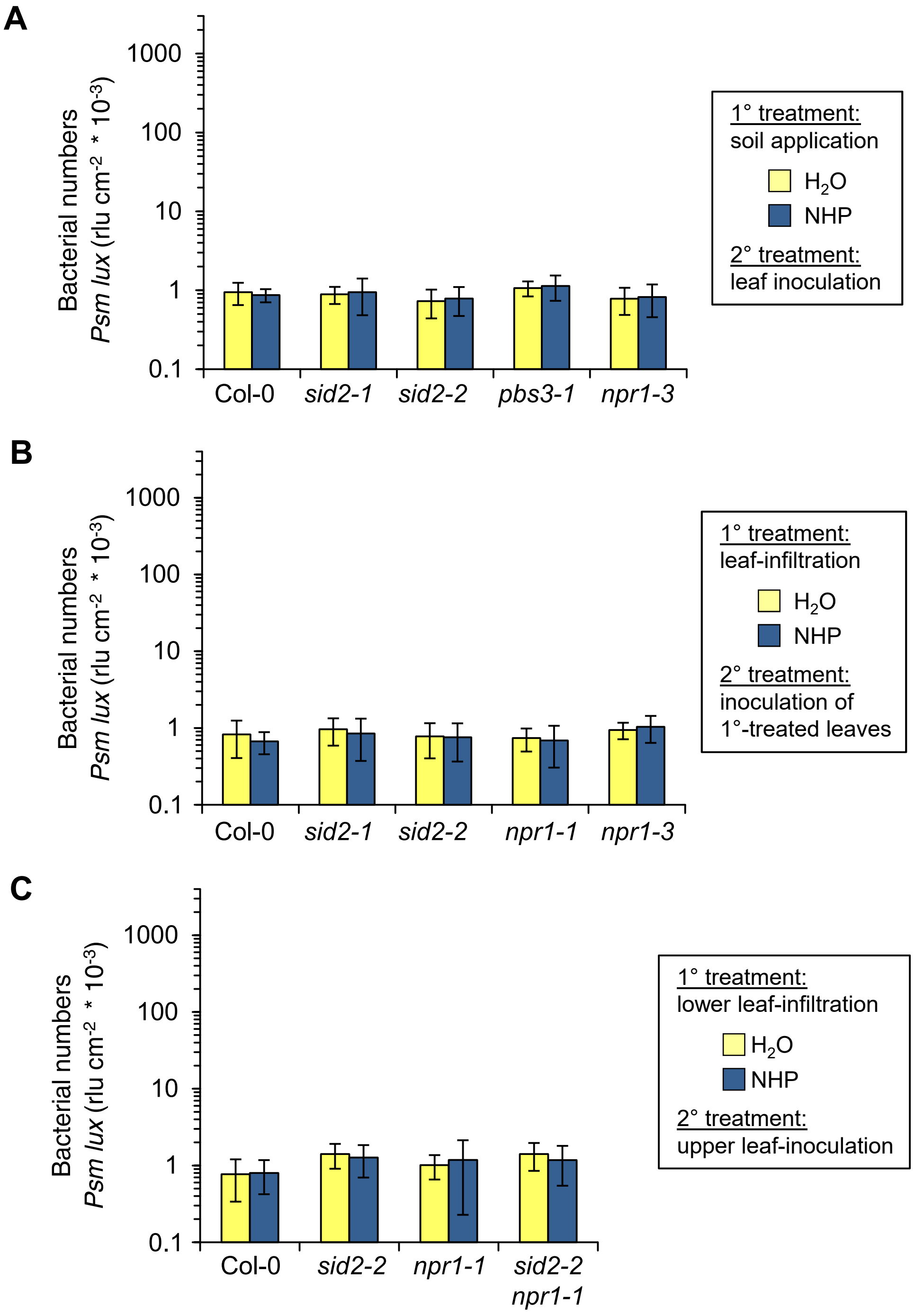

Supplemental Figure S1. Assessment of initial bacterial numbers in leaves after inoculation with *Psm lux* in the experimental settings presented in Fig. 1.

Please refer to Fig. 1 for the experimental details. Two hours after infiltration of bacterial suspensions of *Psm lux*, the initially water-soaked appearance of the leaves had disappeared and the bacterial numbers were quantified by luminescent measurements in order to assess a starting point for the bacterial growth assays. Bars indicate the mean ± SD of at least 9 biological replicates ($n \geq 9$). No statistically significant differences between water- and NHP-pretreated plants were detected (ANOVA and post-hoc Tukey HSD test).

A, NHP (H_2O) pre-treatment via the soil and *Psm lux* inoculation into leaves one day later.

B, NHP (H_2O) infiltration into leaves and *Psm lux* inoculation of the same leaves one day later.

C, NHP (H_2O) infiltration into lower leaves and *Psm lux* inoculation of upper leaves one day later.

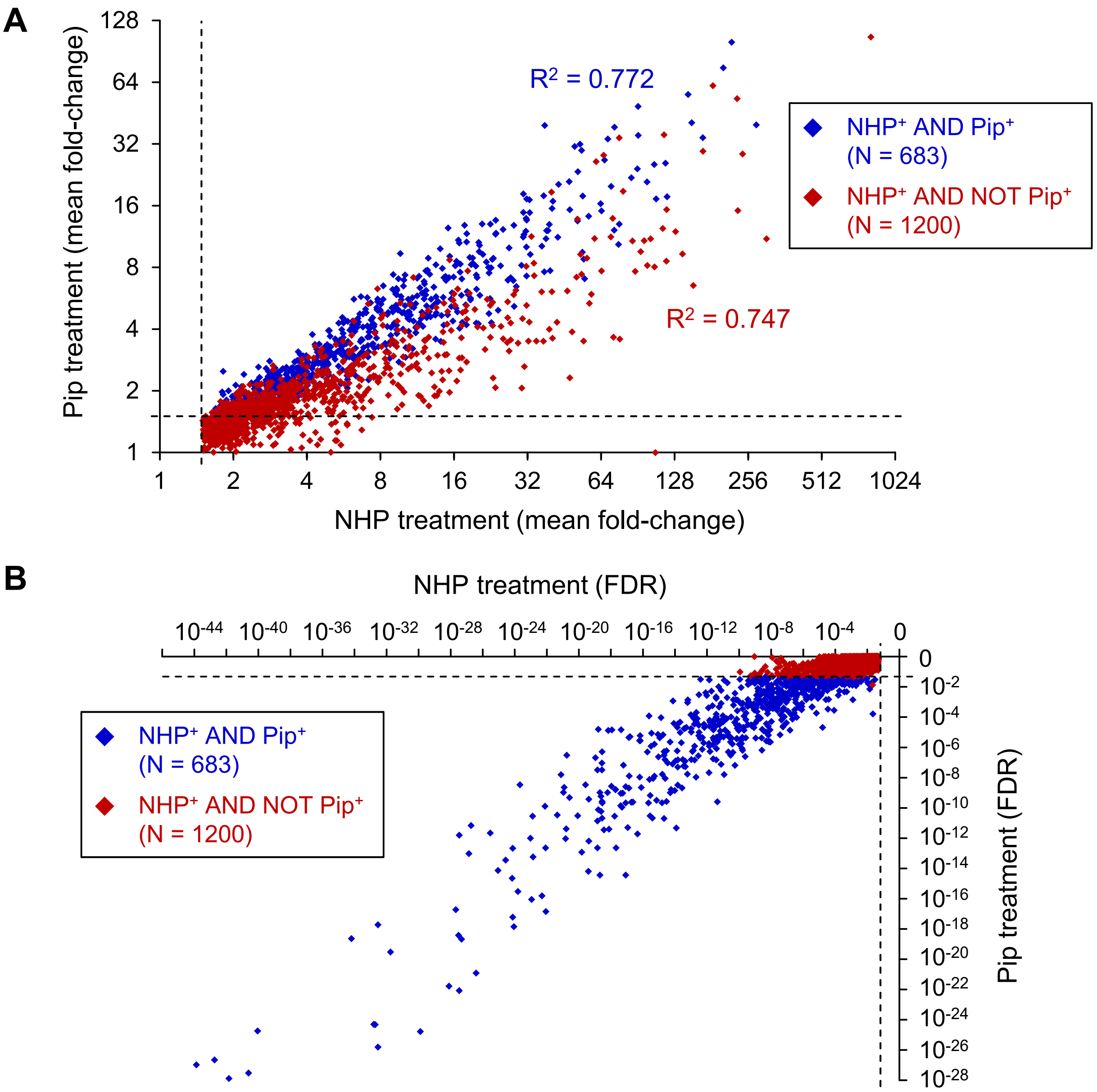

Supplemental Figure S2. The transcriptional response to NHP is qualitatively similar to but quantitatively higher than the transcriptional response to Pip in Col-0 plants.

Comparison of the Col-0 genes that were classified as NHP$^+$ and Pip$^+$ genes (N = 683) with those defined as NHP$^+$ but not Pip$^+$ genes (N = 1200) in the RNA-sequencing analyses depicted in Fig. 3B.

A, The mean values of fold-changes after Pip treatment are plotted against the mean values of fold-changes after NHP treatment for the NHP$^+$ AND Pip$^+$ genes (blue) and the NHP$^+$ AND NOT Pip$^+$ genes (red). The dashed lines indicate the defined gene cutoffs at fold-changes < 1.5 (see method section). In both gene groups, a high linear correlation between the mean fold-changes following NHP- and Pip-treatments was observed. The coefficients of determination (R^2 values), as calculated in Microsoft Excel by the RSQ function for linear correlations, are depicted for both gene groups. Thus, a high proportion of genes from the "NHP$^+$ AND NOT Pip$^+$" group were tendentially up-regulated by Pip$^+$ but were not classified as Pip$^+$ genes because of a variation between the different experiments that resulted in FDR values > 0.05 (see B). The plot also illustrated that the response to NHP was quantitatively higher than the response to Pip. A significant number of genes from the "NHP$^+$ AND NOT Pip$^+$" group were also not up-regulated strongly enough by Pip to make the 1.5-fold change cutoff required for a categorization into the Pip$^+$ gene group.

B, The FDR values calculated from the expression data for the Pip-treated Col-0 samples are plotted against the FDR values for the NHP-treated Col-0 samples, again for both the "NHP$^+$ AND Pip$^+$" genes (blue) and the "NHP$^+$ AND NOT Pip$^+$" genes (red). Nearly all of the "NHP$^+$ AND NOT Pip$^+$" genes showed a FDR > 0.05 for the Pip treatment, indicating that the variation of the Pip-responses in the three independent experiments was the main factor why the were not categorized as Pip$^+$ genes.

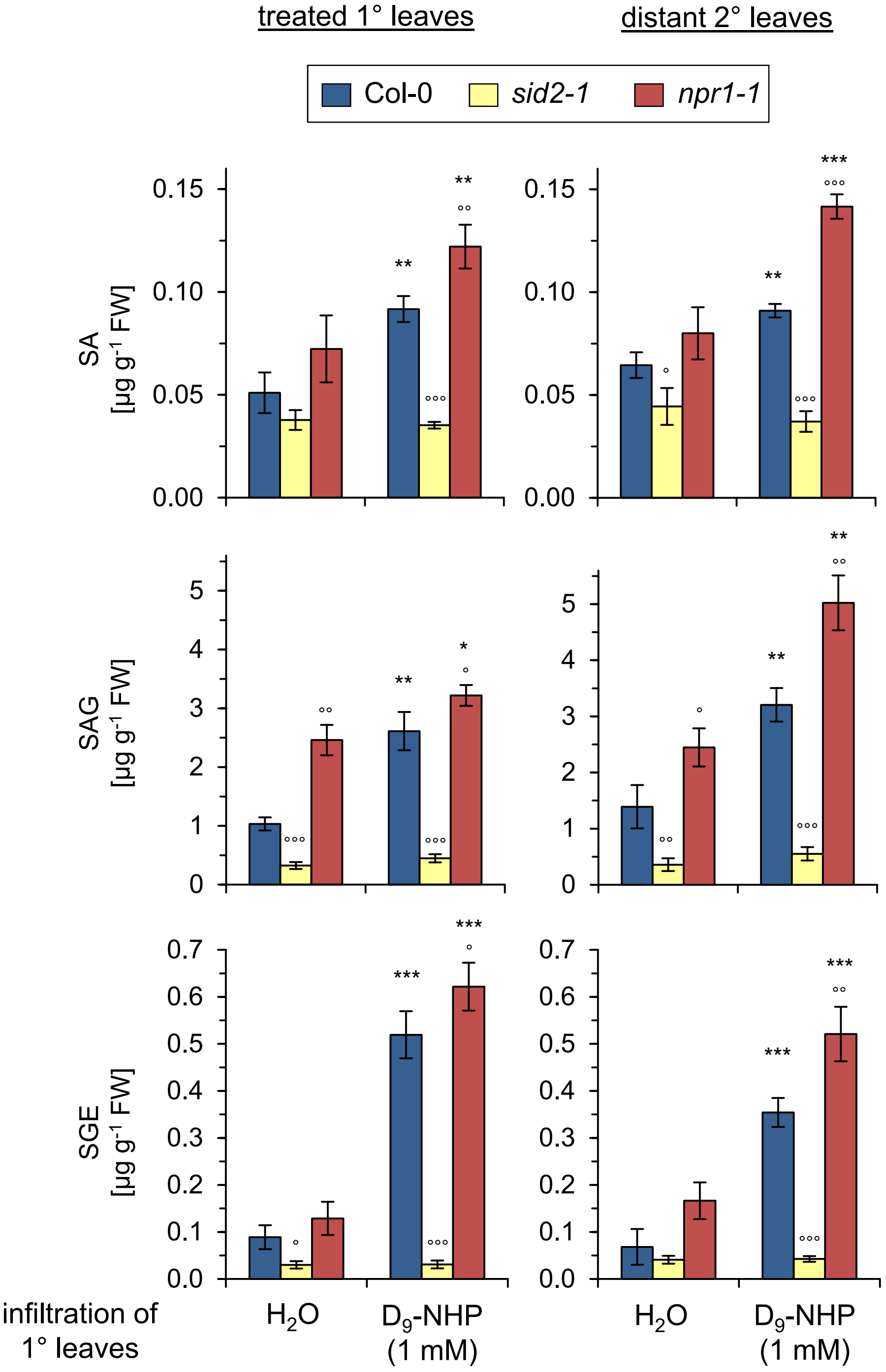

Supplemental Figure S3. Leaf-applied D_9-NHP induces systemic SA accumulation independently from NPR1. Lower, 1° leaves of Arabidopsis Col-0, *sid2-1* or *npr1-1* were infiltrated with 1 mM deuterium-labelled NHP (D_9-NHP), and the contents of free salicylic acid (SA), SA-β-glucoside (SAG) and SA glucose ester (SGE) were determined 24 h later in the 1°-treated and in distant upper (2°) leaves and are given in µg g^{-1} fresh weight (FW). Bars represent means ± SD of four biological replicates (n = 4). Asterisks indicate statistically significant differences between the H_2O-control and the D_9-NHP treatments for a particular genotype (***$P < 0.001$, **$P < 0.01$, *$P < 0.05$; two tailed *t* test). Circles denote statistically significant differences of Col-0 and mutant samples within a same treatment (°°°$P < 0.001$, °°$P < 0.01$, °$P < 0.05$; two tailed *t* test). Related to Fig. 6.

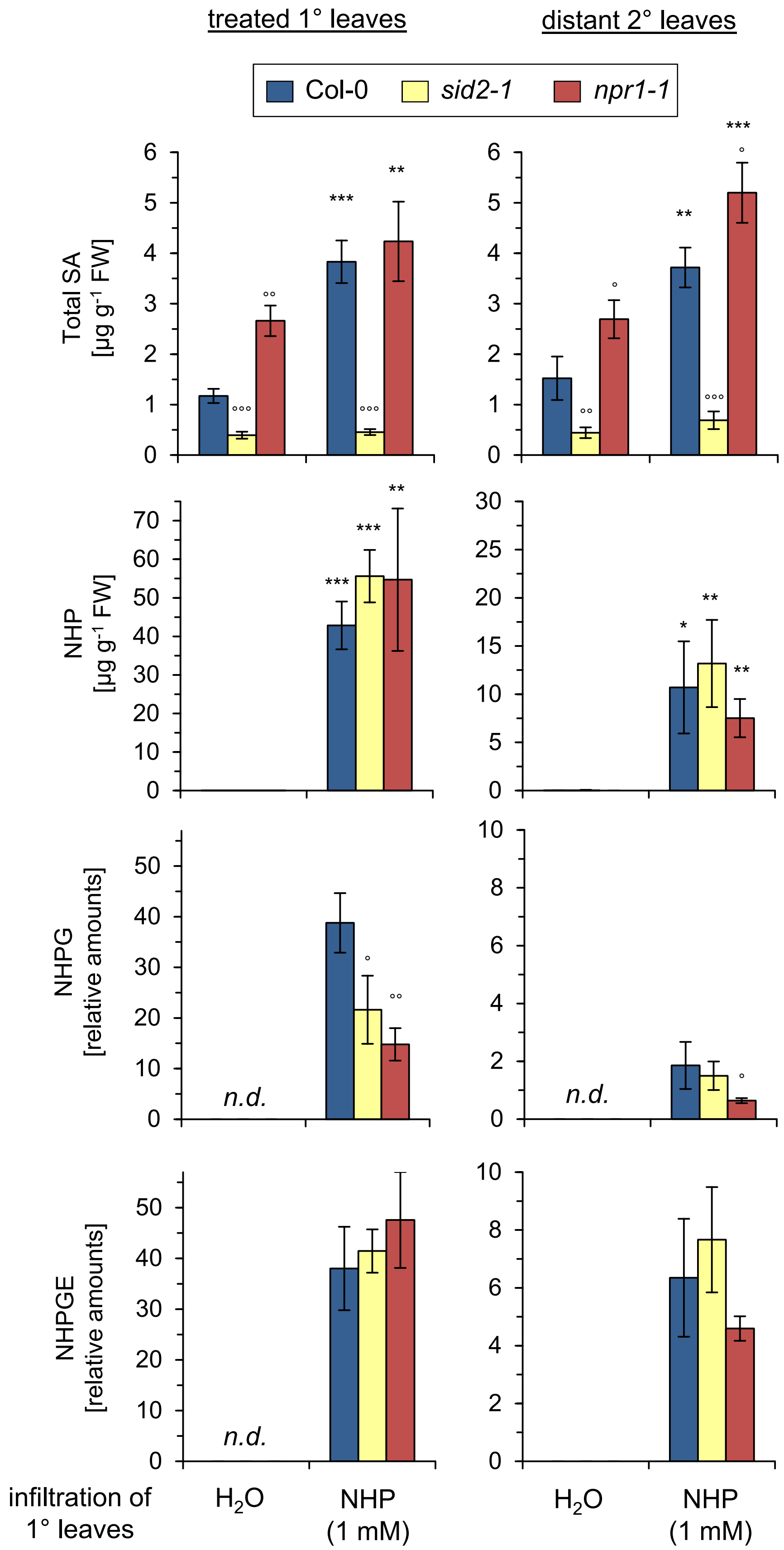

Supplemental Figure S4. Leaf-applied NHP translocates from treated to distant leaves, is partially glycosylated, and induces systemic SA accumulation in an *NPR1*-independent manner.

Lower, 1° leaves of Arabidopsis Col-0, *sid2-1* or *npr1-1* were infiltrated with 1 mM NHP solution, and the contents of NHP, NHP-β-glucoside (NHP-G), NHP-glucose ester (NHP-GE), salicylic acid (SA), SA-β-glucoside and SA glucose ester were determined 24 h later in the 1°-treated and in distant upper (2°) leaves. Total SA represents the sum of unconjugated SA and the two glycosylated SA forms. SA and NHP levels are given in µg g^{-1} FW. NHP-G and NHP-GE are given as relative, FW-related amounts. Bars represent means ± SD of four biological replicates (n = 4). Asterisks indicate statistically significant differences between the H_2O-control and the NHP treatments for a particular genotype ($^{***}P < 0.001$, $^{**}P < 0.01$; $^{*}P < 0.05$ two tailed *t* test). Circles denote statistically significant differences of Col-0 and mutant samples within a same treatment ($^{°°°}P < 0.001$, $^{°°}P < 0.01$, $^{°}P < 0.05$; two tailed *t* test). Related to Fig. 6.

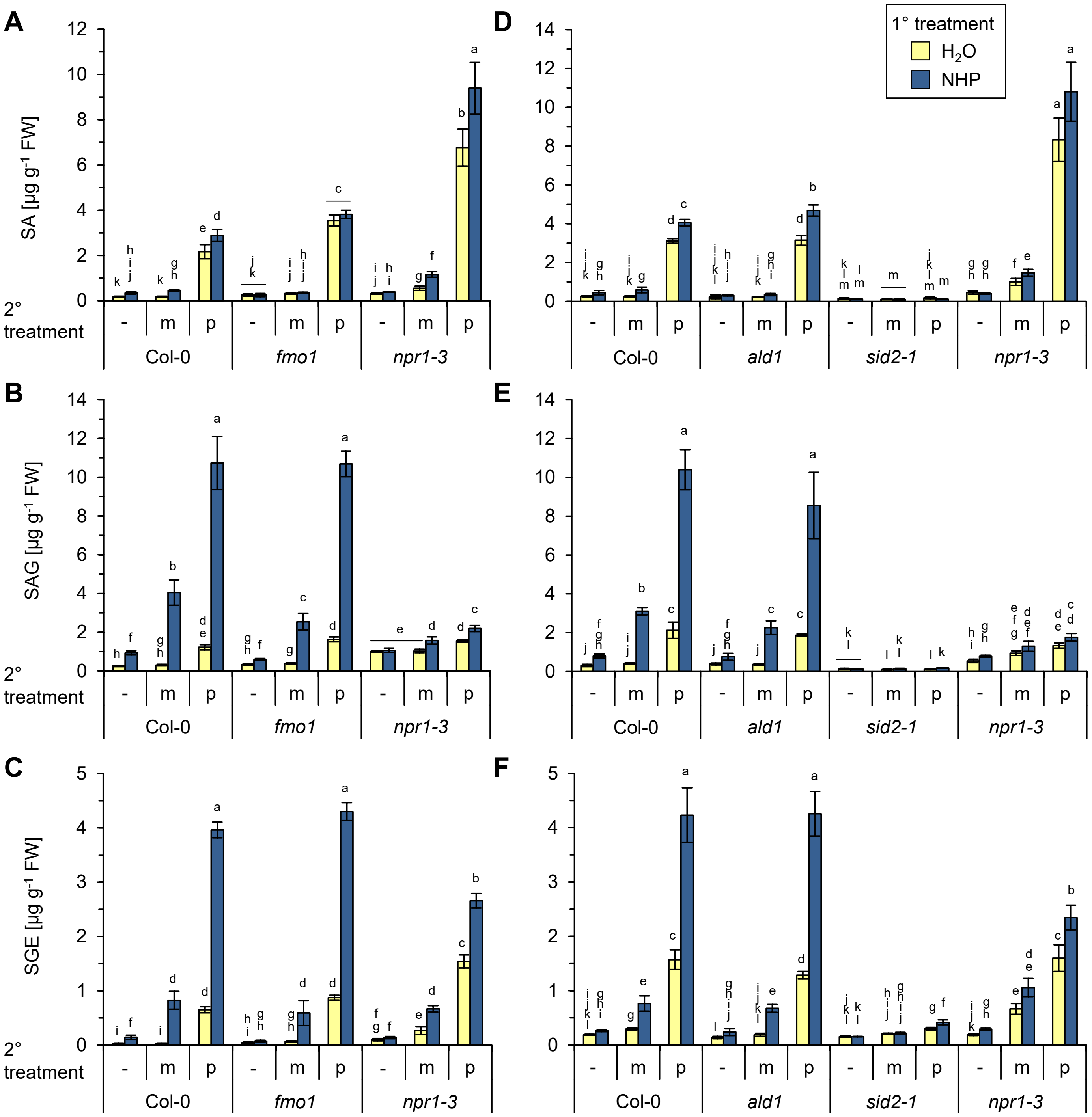

Supplemental Figure S5. Exogenous NHP primes plants for enhanced stimulus-induced salicylic acid biosynthesis.

Plants were watered with 10 ml of 1 mM NHP or 10 ml of H_2O (1° treatment), and leaves challenge-inoculated with *Psm* (p) or mock-infiltrated (m) with 10 mM $MgCl_2$ one day later (2° treatment). The leaves of a third set of plants were left untreated (-). Metabolite levels in leaves were determined 12 h after the 2° treatment. Bars represent means ± SD of four biological replicates (n = 4). Different letters denote significant differences ($p < 0.05$, Kruskal-Wallis H test).

A and D, accumulation of free, unconjugated SA.

B and E, accumulation of SA-β-glucoside (SAG).

C and F, accumulation of SA glucose ester (SGE).

(A) to (C) Experiment with Col-0, *fmo1*, and *npr1-3*. (D) to (F) Independent experiment with Col-0, *ald1*, *sid2-1*, and *npr1-3*. Related to Fig. 8.

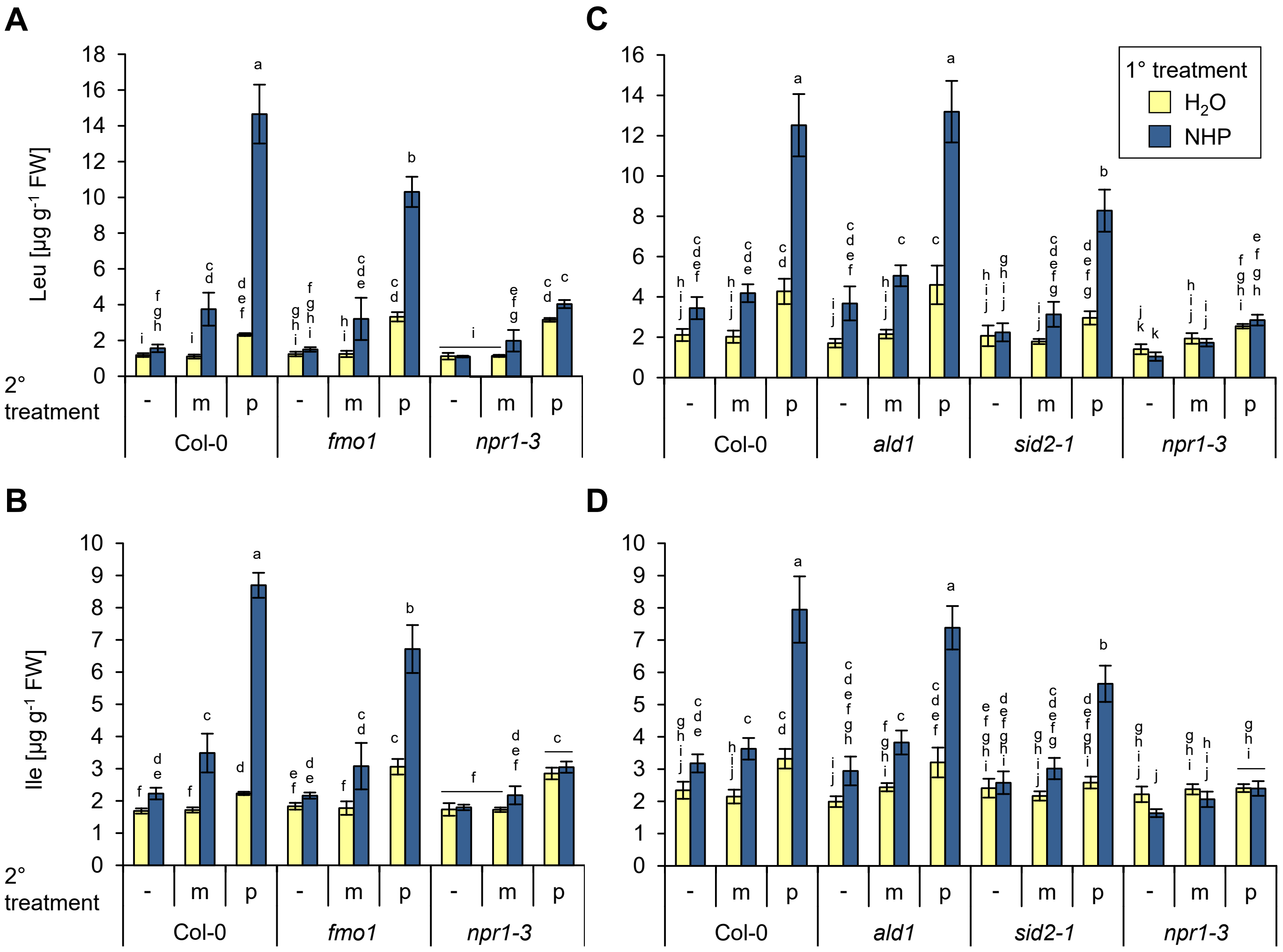

Supplemental Figure S6. Exogenous NHP primes plants for enhanced pathogen-induced branched-chain amino acid accumulation.
A and C, Leucine (Leu) accumulation.
B and D, Isoleucine (Ile) accumulation.
Plants were watered with 10 ml of 1 mM NHP or 10 ml of H_2O (1° treatment), and leaves challenge-inoculated with *Psm* (p) or mock-infiltrated (m) with 10 mM $MgCl_2$ one day later (2° treatment). The leaves of a third set of plants were left untreated (-). Metabolite levels in leaves were determined 12 h after the 2° treatment. Bars represent means ± SD of four biological replicates (n = 4). Different letters denote significant differences ($p < 0.05$, Kruskal-Wallis H test). (A) and (B) Experiment with Col-0, *fmo1*, and *npr1-3*. (C) and (D) Independent experiment with Col-0, *ald1*, *sid2-1*, and *npr1-3*. Related to Fig. 8.

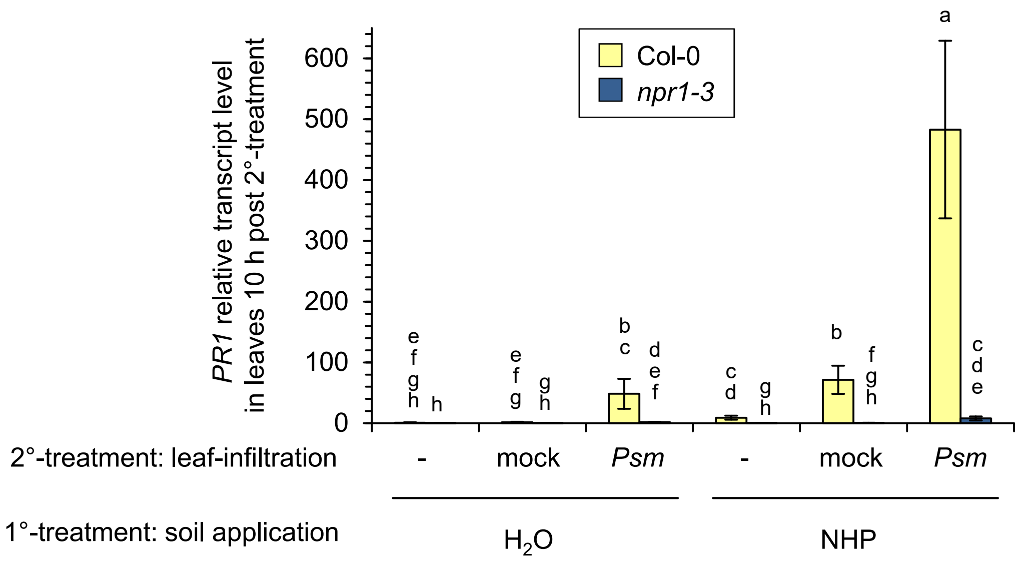

Supplemental Figure S7. NHP applied via the soil primes the Arabidopsis foliage for enhanced *PR1* expression in an NPR1-dependent manner.

Wildtype Col-0 and *npr1-3* plants were supplied with 10 ml of 1 mM NHP or 10 ml of H_2O via the soil (1° treatment), and leaves challenge-inoculated with *Psm* or mock-infiltrated with 10 mM $MgCl_2$ one day later (2° treatment). The leaves of a third set of plants were left untreated (-). The transcript levels of *PR1* in the leaves were determined 10 h after the 2° treatment by qPCR analysis. Transcript values are given relative to the mean value of the Col-0 control samples (1° treatment H_2O / no 2° treatment). Bars indicate the mean ± SD of 4 biological replicates (n = 4). Different letters denote significant differences ($p < 0.05$, Kruskal-Wallis H test). Related to Fig. 9.

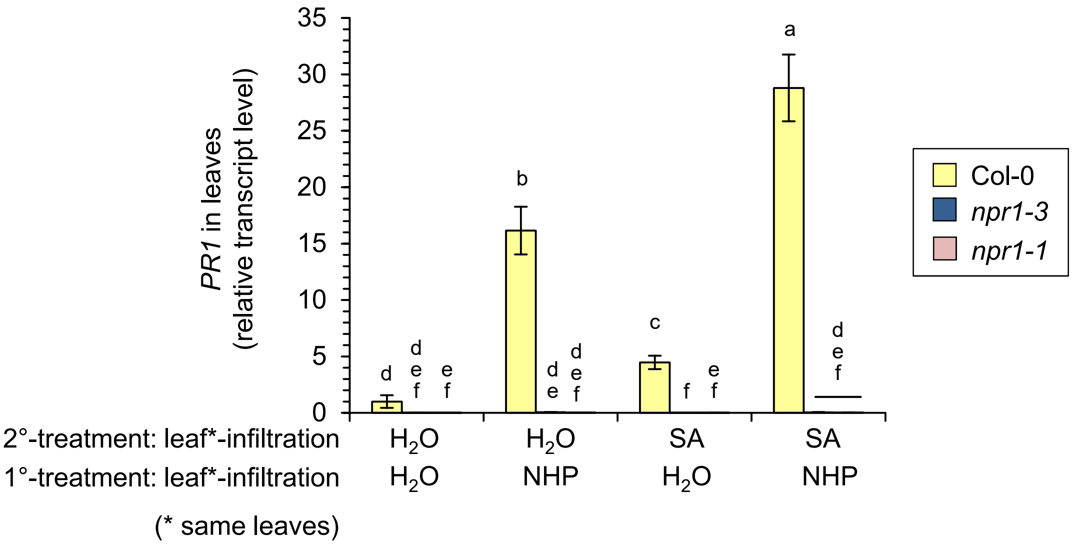

Supplemental Figure S8. Arabidopsis leaves treated with exogenous NHP are primed for enhanced SA-inducible *PR1* expression.

Three leaves of Col-0, *npr1-1*, or *npr1-3* plants were infiltrated with 1 mM NHP (or H_2O) (1° treatment) and the same leaves infiltrated one day later with 0.5 mM SA (or H_2O) (2 ° treatment). Leaf *PR1* transcript levels were determined 4 h after the 2° treatment (n = 4) and are given relative to the mean of the 1°-H_2O- and 2°-H_2O-treated samples.

Wildtype Col-0 and *npr1-3* plants were supplied with 10 ml of 1 mM NHP or 10 ml of H_2O via the soil (1° treatment), and leaves challenge-inoculated with *Psm* or mock-infiltrated with 10 mM $MgCl_2$ one day later (2° treatment). The leaves of a third set of plants were left untreated (-). The transcript levels of *PR1* in the leaves were determined 10 h after the 2° treatment by qPCR analysis. Transcript values are given relative to the mean value of the Col-0 control samples (1° treatment H_2O / no 2° treatment). Bars indicate the mean ± SD of 4 biological replicates (n = 4). Different letters denote significant differences ($p < 0.05$, Kruskal-Wallis H test). Related to Fig. 9.

File name (FastQ)	Sample ID	Genotype	Treat-ment	Experi-ment	Replicate	Harvest	N° of leaves (Leaves x plant)
01_Col0_H_Exp1_R1	1	Col-0	H_2O	Exp1	R1	24 h	18 (3 x 6)
02_Col0_H_Exp2_R2	2	Col-0	H_2O	Exp2	R2	24 h	18 (3 x 6)
03_Col0_H_Exp3_R3	3	Col-0	H_2O	Exp3	R3	24 h	18 (3 x 6)
04_Col0_N_Exp1_R1	4	Col-0	NHP	Exp1	R1	24 h	18 (3 x 6)
05_Col0_N_Exp2_R2	5	Col-0	NHP	Exp2	R2	24 h	18 (3 x 6)
06_Col0_N_Exp3_R3	6	Col-0	NHP	Exp3	R3	24 h	18 (3 x 6)
07_Col0_P_Exp1_R1	7	Col-0	Pip	Exp1	R1	24 h	18 (3 x 6)
08_Col0_P_Exp2_R2	8	Col-0	Pip	Exp2	R2	24 h	18 (3 x 6)
09_Col0_P_Exp3_R3	9	Col-0	Pip	Exp3	R3	24 h	18 (3 x 6)
10_npr1_H_Exp1_R1	10	*npr1-3*	H_2O	Exp1	R1	24 h	18 (3 x 6)
11_npr1_H_Exp2_R2	11	*npr1-3*	H_2O	Exp2	R2	24 h	18 (3 x 6)
12_npr1_H_Exp3_R3	12	*npr1-3*	H_2O	Exp3	R3	24 h	18 (3 x 6)
13_npr1_N_Exp1_R1	13	*npr1-3*	NHP	Exp1	R1	24 h	18 (3 x 6)
14_npr1_N_Exp2_R2	14	*npr1-3*	NHP	Exp2	R2	24 h	18 (3 x 6)
15_npr1_N_Exp3_R3	15	*npr1-3*	NHP	Exp3	R3	24 h	18 (3 x 6)
16_sid2_H_Exp1_R1	16	*sid2-1*	H_2O	Exp1	R1	24 h	18 (3 x 6)
17_sid2_H_Exp2_R2	17	*sid2-1*	H_2O	Exp2	R2	24 h	18 (3 x 6)
18_sid2_H_Exp3_R3	18	*sid2-1*	H_2O	Exp3	R3	24 h	18 (3 x 6)
19_sid2_N_Exp1_R1	19	*sid2-1*	NHP	Exp1	R1	24 h	18 (3 x 6)
20_sid2_N_Exp2_R2	20	*sid2-1*	NHP	Exp2	R2	24 h	18 (3 x 6)
21_sid2_N_Exp3_R3	21	*sid2-1*	NHP	Exp3	R3	24 h	18 (3 x 6)

Supplemental Table S1. Experimental setup to examine the transcriptional response to NHP by RNA-sequencing.

To investigate the transcriptional response of Arabidopsis wild-type plants (Col-0) and selected mutants (*npr1-3* and *sid2-1*) to elevated NHP (or Pip), individual, 5-week-old soil-grown plants were supplied via the root with 10 µmol NHP, 10 µmol Pip (only in the case of Col-0 plants), or treated with an equivalent volume of water, which served as solvent and thus control treatment. 18 full-grown leaves from 6 different plants (3 leaves per plant) were harvested 24 h after the respective treatment and pooled to generate one biological replicate sample. In total, three biologically independent, replicate experiments were performed. As a result, three biologically independent samples per treatment and plant genotype were obtained. RNA-seq analysis of the resulting 21 samples was performed as described in the main text. The respective sample ID of each replicate is indicated in the table and matches the file annotations of the raw data files (FastQ format) deposited to ArrayExpress.

	gene category	number of genes in			% gene category in			fold-enrichment	
		genome	NHP^+	SAR^+	genome	NHP^+	SAR^+	NHP^+	SAR^+
**A**	<u>GO term analyses</u>								
	total number of genes	27416	1854	3027	-	-	-	-	-
	response to stress	3079	472	730	11.2	25.4	24.1	2.3^{***}	2.2^{***}
	immune system process	372	109	152	1.4	5.9	5.0	4.3^{***}	3.7^{***}
	cell communication	1560	218	335	5.7	11.8	11.1	2.1^{***}	2.0^{***}
	small molecule binding	2133	256	424	7.8	13.8	14.0	1.8^{***}	1.8^{***}
	protein glycosylation	104	26	24	0.4	1.4	0.8	3.7^{***}	2.1
	plasma membrane	3791	484	763	13.8	26.1	25.2	1.9^{***}	1.8^{***}
	endomembrane system	2391	306	557	8.7	16.5	18.4	1.9^{***}	2.1^{***}
	secretory vesicle	166	36	41	0.6	1.9	1.4	3.2^{***}	2.2^{*}
	endoplasmic reticulum	912	162	244	3.3	8.7	8.1	2.6^{***}	2.4^{***}
	Golgi apparatus	1219	144	282	4.5	7.8	9.3	1.8^{***}	2.1^{***}
	vacuole	1135	126	205	4.1	6.8	6.8	1.6^{***}	1.6^{***}
	leaf senescence	103	23	37	0.4	1.2	1.2	3.3^{***}	3.3^{***}
	catabolic process	1488	135	269	5.4	7.3	8.9	1.3	1.6^{***}
	transport	2086	173	378	7.6	9.3	12.5	1.2	1.6^{***}
	anatomical development	2885	158	337	10.5	8.5	11.1	0.8	1.1
	biosynthetic process	2441	143	251	8.9	7.7	8.3	0.9	0.9
	nucleic acid metabolism	1629	54	90	5.9	2.9	3.0	0.5^{***}	0.5^{***}
	translation	431	8	19	1.6	0.4	0.6	0.3^{*}	0.4^{*}
	organelle organization	1608	65	172	5.9	3.5	5.7	0.6^{*}	1.0
	reproductive development	1192	44	108	4.4	2.4	3.6	0.6^{*}	0.8
**B**	<u>family analyses</u>								
	total number of genes	26711	1870	3061	-	-	-	-	-
	PDIL	22	6	7	0.08	0.32	0.23	3.9^{**}	2.8^{*}
	SPFH	10	5	9	0.04	0.27	0.29	7.1^{**}	7.9^{***}
	VQ	34	8	13	0.13	0.43	0.42	3.4^{**}	3.3^{***}
	FAX	7	4	4	0.03	0.21	0.13	8.2^{**}	5.0^{*}
	PUB	60	10	20	0.22	0.53	0.65	2.4^{*}	2.9^{***}
	ZAT	20	6	7	0.07	0.32	0.23	4.3^{**}	3.1^{*}
	HIPP	44	8	8	0.16	0.43	0.26	2.6^{*}	1.6
	GST	52	8	19	0.19	0.43	0.62	2.2	3.2^{***}
	NUDIX	28	4	8	0.10	0.21	0.26	2.0	2.5^{*}
	PCR	12	3	3	0.04	0.16	0.10	3.6	2.2
	UGT	117	13	15	0.44	0.70	0.49	1.6	1.1
	CYP450	244	16	25	0.91	0.86	0.82	0.9	0.9
	PER	73	5	7	0.27	0.27	0.23	1.0	0.8
	MYB	150	5	9	0.56	0.27	0.29	0.5	0.5

Supplemental Table S2. Occurrence of NHP^+ and SAR^+ genes in further groups of Gene Ontology (GO) terms and gene families.

A, NHP^+ and SAR^+ genes in distinct GO term categories (https://www.arabidopsis.org/tools/go_term_enrichment.jsp). The first row depicts the total

number of genes in the reference list of the TAIR gene enrichment tool and the numbers of NHP^+ and SAR^+ genes in this list (see also Fig. 3). The other rows depict the absolute number of genes of a particular GO category in the whole genome, in the NHP^+ group and in the SAR^+ group (left columns), the percentages of genes from the GO categories in the whole genome, NHP^+ and SAR^+ groups (middle columns), and the fold-enrichment of the NHP^+- and SAR^+ gene groups with respect to the whole genome (asterisks indicate significant enrichment or depletion; Fisher's exact test; *: $P<0.05$, **: $P< 0.001$, ***: $P< 0.0001$).

B, Gene family analysis based on TAIR10 family annotation and published lists of gene families. The total genes used for family analysis (26711) comprised the merged list of total genes covered in both the NHP- and SAR-related RNA-seq analyses (Fig. 3C). See (A) for further information. Abbreviations: PDIL: protein disulfide isomerase-like; SPFH: SPFH/Band 7/PHB domain-containing membrane-associated protein family; VQ: VQ motif-containing proteins; FAX: fatty acid export; PUB: U-box domain-containing protein family; ZAT: subclass C1-2i of Cys2His2 (C2H2)-type zinc-finger protein (ZFP) family; HIPP: heavy metal-associated isoprenylated plant proteins; GST: glutathione-S-transferases; NUDIX: nudix hydrolase homologs; PCR: PLANT CADMIUM RESISTANCE; UGT: UDP-dependent glycosyltransferases; CYP450: cytochrome P450 superfamily; PER: peroxidase superfamily proteins; MYB: MYB domain transcription factors.

Related to Table 1.

	Name	AGI Code	Gene Name / Description	NHP / H_2O fold-change
A	*ALD1*	At2g13810	AGD2-LIKE DEFENSE RESPONSE PROTEIN 1	88.3
	SARD4	At5g52810	SAR DEFICIENT 4	5.5
	FMO1	At1g19250	FLAVIN-DEPENDENT MONOOXYGENASE 1	34.1
	UGT76B1	At3g11340	UDP-DEPENDENT GLYCOSYLTRANSFERASE 76B1	99.0
B	*ICS1*	At1g74710	ISOCHORISMATE SYNTHASE 1	4.9
	EDS5	At4g39030	ENHANCED DISEASE SUSCEPTIBILITY 5	5.1
	PBS3	At5g13320	AVRPPHB SUSCEPTIBLE 3	10.7
	S3H	At4g10500	SALICYLIC ACID-3-HYDROXYLASE	105.7
	S5H	At5g24530	SALICYLIC ACID-5-HYDROXYLASE	25.4
	UGT74F1	At2g43840	UDP-DEPENDENT GLYCOSYLTRANSFERASE 74F1	0.4
	UGT74F2	At2g43820	UDP-DEPENDENT GLYCOSYLTRANSFERASE 74F2	2.9
C	*EDS1*	At3g48090	ENHANCED DISEASE SUSCEPTIBILITY 1	5.9
	PAD4	At3g52430	PHYTOALEXIN DEFICIENT 4	7.1
	SAG101	At5g14930	SENESCENCE-ASSOCIATED GENE 101	3.0
	SARD1	At1g73805	SAR DEFICIENT 1	10.5
	CBP60G	At5g26920	CALM-BINDING PROTEIN 60-LIKE G	6.0
D	*NPR1*	At1g64280	NONEXPRESSER OF PR GENES 1	2.7
	NPR3	At5g45110	NPR1-LIKE PROTEIN 3	4.0
	NPR4	At4g19660	NPR1-LIKE PROTEIN 4	3.3
	NIMIN1	At1g02450	NIM1-INTERACTING 1	167.1
	NIMIN2	At3g25882	NIM1-INTERACTING 2	117.5
	MLO2	At1g11310	MILDEW RESISTANCE LOCUS O2	3.7
E	*PR1*	At2g14610	PATHOGENESIS-RELATED GENE 1	114.5
	PR2	At3g57260	PATHOGENESIS-RELATED GENE 2	27.3
	PR5	At1g75040	PATHOGENESIS-RELATED GENE 5	9.9
F	*CRK7*	At4g23150	CYSTEINE-RICH RECEPTOR-LIKE KINASE7	30.3
	CRK37	At5g26920	CYSTEINE-RICH RECEPTOR-LIKE KINASE37	22.3
	MPK3	At3g45640	MITOGEN-ACTIVATED PROTEIN KINASE 3	2.9
	MPK11	At1g01560	MITOGEN-ACTIVATED PROTEIN KINASE 11	18.7
	CPK5	At4g35310	CALCIUM-DEPENDENT PROTEIN KINASE5	2.3
	CPK6	At5g26920	CALCIUM-DEPENDENT PROTEIN KINASE6	2.3
	WRKY33	At2g38470	WRKY domain transcription factor 33	5.2
	WRKY54	At2g40750	WRKY domain transcription factor 54	52.5
G	*NDR1*	At3g20600	NON RACE-SPECIFIC DISEASE RESISTANCE 1	7.1
	ACD6	At4g14400	ACCELERATED CELL DEATH 6	9.0
	ATG2	At3g19190	AUTOPHAGY2	2.9
	MC2	At4g25110	METACASPASE 2	14.1
	MC8	At1g16420	METACASPASE 8	19.7
	HIR2	At1g69840	HYPERSENSITIVE INDUCED REACTION 2	2.6
	HIR3	At3g01290	HYPERSENSITIVE INDUCED REACTION 3	11.2
H	*SYP121*	At3g11820	SYNTAXIN OF PLANTS 121	4.0
	SYP122	At3g52400	SYNTAXIN OF PLANTS 122	7.3
	SNAP33	At5g61210	sol. N-ethylmaleimide-sensitive factor adaptor protein 33	3.2
	PEN3	At1g59870	PENETRATION 3	4.6
	CALS1	At1g05570	CALLOSE SYNTHASE 1	6.4
I	*CYP79B2*	At4g39950	Cytochrome P450 79B2	0.7
	CYP79B3	At2g22330	Cytochrome P450 79B3	0.7
	CYP71A12	At2g30750	Cytochrome P450 71A12	4.7
	CYP71A13	At2g30770	Cytochrome P450 71A13	16.2
	GGP1	At4g30530	GAMMA-GLUTAMYL PEPTIDASE 1	0.8
	PAD3	At3g26830	PHYTOALEXIN DEFICIENT 3 / CYP71B15	26.6

Supplemental Table S3. Selected immune-related genes upregulated by NHP.

The table extends the information of Figure 4. Exemplary defense-related genes from different categories out of the 1883 NHP$^+$ genes determined by RNA-seq analysis (Fig. 3) are depicted.

A, NHP biosynthesis and metabolism.

B, SA biosynthesis and metabolism.

C, Regulation of SA and NHP biosynthesis.

D, SA/NHP downstream signaling.

E, Pathogenesis-related genes.

F, Immune-related signal transduction.

G, Genes related to hypersensitive cell death.

H, Cell-wall based defense and non-host resistance.

I, Camalexin biosynthesis.

The right column depicts the ratios of the mean expression values of NHP- vs. water-control-samples. The colouring is according to the heat map shown in Fig. 4. A few depicted genes do not belong to the NHP$^+$ gene group (grey background).

gene category	number of genes in			% GO term genes in			fold-enrichment	
	genome	NHP^-	SAR^-	genome	NHP^-	SAR^-	NHP^-	SAR^-
<u>GO term analyses</u>								
total number of genes	27416	657	2832	-	-	-	-	-
metabolic process	7731	186	1084	28.2	28.3	38.3	1.0	1.4***
glucose metabolism	44	4	19	0.2	0.6	0.7	3.8	4.2**
α-amino acid biosynthesis	162	6	57	0.6	0.9	2.0	1.6	3.4***
fatty acid metabolism	201	13	57	0.7	2.0	2.0	2.7	2.8***
chromosome organization	512	2	15	1.9	0.3	0.5	0.2	0.3***
ubiquitin-dep. protein catabolism	480	5	15	1.8	0.8	0.5	0.4	0.3***
ribonucleoprotein complex	711	2	68	2.6	0.3	2.4	0.1**	0.9
gene expression	1403	9	168	5.1	1.4	5.9	0.3**	1.2

Supplemental Table S4. Occurrence of NHP^- and SAR^- genes in further groups of Gene Ontology (GO) terms.

See Supplemental Table S2 for further details. Related to Table 2.

A	Gene		Primer sequence (5' to 3')	
	At2g13810	*ALD1*	Forward primer<br>Reverse primer	GTGCAAGATCCTACCTTCCCGGC<br>CGGTCCTTGGGGTCATAGCCAGA
	At2g30770	*CYP71A13*	Forward primer<br>Reverse primer	ACATAGTTGAATCTTTCCCAAAGCACAAT<br>CAAATTACCGGGATCTCGAACGGTGG
	At1g19250	*FMO1*	Forward primer<br>Reverse primer	TCTTCTGCGTGCCGTAGTTTC<br>CGCCATTTGACAAGAAGCATAG
	At1g74710	*ICS1*-B	Forward primer<br>Reverse primer	TTCTGGGCTCAAACACTAAAAC<br>GGCGTCTTGAAATCTCCATC
	At1g74710	*ICS1*-C	Forward primer<br>Reverse primer	ATGCGGGGACAGGGATAGTAG<br>TCGCCTGTAGAGATGTTGTTGC
	At3g26830	*PAD3*	Forward primer<br>Reverse primer	GGCTGAAGCGGTCATAAGAG<br>TCCAGGCTTAAGATGCTCGT
	At5g13320	*PBS3*	Forward primer<br>Reverse primer	TGCCTGCTCGAGTCGCAACC<br>TGGACTAAGCCACAGAGCAAATGGC
	At2g14610	*PR1*	Forward primer<br>Reverse primer	GTGCTCTTGTTCTTCCCTCG<br>GCCTGGTTGTGAACCCTTAG
	At1g43190	*PTB*	Forward primer<br>Reverse primer	GATCTGAATGTTAAGGCTTTTAGCG<br>GGCTTAGATCAGGAAGTGTATAGTCTCTG
	At3g11340	*UGT76B1*	Forward primer<br>Reverse primer	CTTTACAAGAGACTAAGGCAG<br>CACACCTATCTGTAACTTATCCC
**B**	**Primer name**		**Primer sequence (5' to 3')**	
	sid2-2-F<br>*sid2-2*-R		CTCAATTAGGTGTCTGCAGTGAAGC<br>GTTGTAGCAAAAACCGTAATGATCG	
	npr1-1-NlaIII-F<br>*npr1-1*-NlaIII-R		AGGCACTTGACTCGGATGAT<br>ATGCACTTGCACC-TTTTTCC	

Supplemental Table S5. Primers used in this study.

A, Primers used for RT-qPCR-analyses.

B, Primers used for identification of the *sid2-2 npr1-1* double mutant.
